# Metabodeconplus—An R Package for Automated Deconvolution and Alignment of 1D NMR Metabolomics Data

**DOI:** 10.3390/metabo16090604

**Published:** 2026-08-24

**Authors:** Tobias Schmidt, Maximilian Sombke, Helena U. Zacharias, Peter J. Oefner, Rainer Spang, Wolfram Gronwald

**Affiliations:** 1 Institute of Statistical Bioinformatics, University of Regensburg, 93053 Regensburg, Germany; tobias2.schmidt@ukr.de (T.S.); rainer.spang@ur.de (R.S.); 2 Institute of Functional Genomics, University of Regensburg, 93053 Regensburg, Germany; maximilian.sombke@stud.uni-regensburg.de (M.S.); peter.oefner@ur.de (P.J.O.); 3 Peter L. Reichertz Institute for Medical Informatics of TU Braunschweig and Hannover Medical School, Hannover Medical School, 30625 Hannover, Germany; helena.zacharias@plri.de

**Keywords:** 1D NMR, deconvolution, metabolites, quantification, R package, signal identification

## Abstract

**Background:** In one-dimensional NMR spectra of complex biofluids such as urine and plasma, extensive signal overlap obscures individual metabolite signals. Resolving this overlap by deconvolution is only the first step: turning a set of spectra into a table for subsequent statistical analysis also requires the alignment of signals across samples and their integration into a single feature matrix. **Methods:** Here, metabodeconplus is presented, an R package that unifies this entire path into a single reproducible end-to-end workflow. From raw one-dimensional spectra, it deconvolutes overlapping signals, aligns resulting signals across samples, and integrates them into a data matrix for built-in sample classification or downstream statistical analysis. Automated parameter optimization removes manual tuning, and a Rust computational backend with parallelization leads to fast runtimes. **Results:** On the simulated Sim3 spectra, a combined score of correctly identified signals and reconstruction accuracy (maximum 1) rose from 0.712 for the predecessor package to 0.801 for metabodeconplus. For the urinary AKI dataset, metabodeconplus reached a classification accuracy of 73.7 ± 2.20% and an AUC=0.827±0.025, which is comparable to the binning baseline. An advantage is the potential unambiguous metabolite assignment of the deconvoluted signals. **Conclusions:** The package is freely available as open source on GitHub and on CRAN.

## 1. Introduction

### 1.1. Background

Nuclear magnetic resonance (NMR) spectroscopy is a widely used analytical technique in metabolomics [1,2,3]. By exploiting the magnetic properties of atomic nuclei, NMR provides structural and quantitative information about the metabolite composition of biomedical specimens such as urine and plasma, as well as cells and tissues. Alongside mass spectrometry, this makes it a key tool in metabolomics, where researchers seek to characterize the metabolite profile of biological systems.

### 1.2. Challenges in 1D NMR Spectra Analysis

The goal of a metabolomics experiment is to identify and quantify the metabolites present in a sample. To this end, certain physical properties are recorded, allowing inferences about the contained metabolites. In NMR spectroscopy, the measured properties are the resonance frequencies of atomic nuclei like ^1^H in a magnetic field, which are influenced by their electronic environment, and the intensities of these resonances. Following Fourier transformation of the raw time-domain data, the obtained NMR spectrum can be used to infer the presence and concentrations of specific metabolites [4]. However, despite its advantages, NMR spectroscopy faces challenges that complicate the direct extraction of metabolite concentrations from raw spectra. Two major issues that hinder accurate identification and quantification of metabolites are signal overlap and signal shifts.

#### 1.2.1. Signal Overlap

Ideally, the resonance frequencies in an NMR spectrum would appear as sharp signals, allowing straightforward identification and quantification. However, in reality, these signals possess distinct linewidths and, therefore, may overlap with one another, rendering it difficult to distinguish individual signals. Several factors contribute to signal broadening, including $T_{2}$ relaxation effects [5], magnetic field inhomogeneities [6], chemical exchange processes [7], sample viscosity and molecular interactions, which both contribute to relaxation [8], and the measurement process itself.

The process of reconstructing the individual metabolite contributions from a complex spectrum of overlapping signals is known as deconvolution. This is one of the key challenges that metabodeconplus seeks to address.

#### 1.2.2. Signal Shifts

Another major challenge in NMR-based metabolomics is that the resonance frequencies of the same metabolite might slightly vary across samples. Several factors can cause these signal shifts, including variations in temperature [4], differences in sample pH [8], and variations in ionic strength and solvent composition [8]. With respect to variations in pH, pronounced signal shifts are, for example, commonly observed for side-chain resonances in metabolites like histidine, where the pKa of the side-chain is close to physiological levels (approximately 6.0 for histidine).

These inconsistencies complicate direct comparisons between samples and hinder both identification and quantification of metabolites. To address this issue, signal alignment techniques are used to shift signals across spectra to concordant positions, allowing for more accurate quantitative comparisons.

### 1.3. Existing Solutions

Available open-source solutions include the R packages BATMAN [9], rDolphin [10], speaq 2.0 [11], ASICS [12], SigMa [13], and MetaboDecon1D [14] (the predecessor of this package); the Python program decon1d [15]; the MATLAB applications icoshift [16] and COW [17]; and the web applications BAYESIL [18], DEEP Picker1D [19], and NMRProcFlow [20]. Proprietary software suites available for NMR spectral analysis include NMR Workbook Suite by ACD/Labs, AMIX by Bruker, Chenomx NMR Suite by Chenomx Inc., and Mnova NMR by Mestrelab Research. Furthermore, Bruker TopSpin 4.2 includes the program mldecon [21].

A summary of the main features of each tool is provided in Table 1; detailed descriptions of individual solutions are given in Appendix A.

### 1.4. The Role of Metabodeconplus

The aim of metabodeconplus is to deconvolute 1D NMR spectra of complex biofluids such as plasma, urine, or CSF containing numerous overlapping signals to determine accurate integrals of the underlying signals regardless of whether the corresponding metabolites are known. Therefore, no library of known reference compounds is required. As a result, unidentified signals can also be used for a variety of tasks such as sample classification. Previously, MetaboDecon1D [14] has been developed, an R package for the deconvolution and integration of overlapping 1D NMR signals. For each spectrum, the output is a list of deconvoluted signals characterized by their spectral positions together with accurate integral values. However, to make full use of this data, additional signal alignment is required for subsequent statistical analyses. metabodeconplus is not a new version of MetaboDecon1D but a new and substantially broader package: whereas MetaboDecon1D performed deconvolution of a single spectrum, metabodeconplus provides a complete, automated pipeline that takes a set of raw 1D NMR spectra all the way to an aligned, classification-ready table of signal integrals. As deconvoluted signals, in contrast to bins, correspond to single compounds, this also facilitates subsequent potential identification and accurate quantification of compounds by other methods. Note that tools for the latter two tasks are not part of metabodeconplus. For signal alignment, the CluPA algorithm [22] of the speaq 2.0 package [11] was included. To this end, CluPA was adapted to the output format of the deconvolution step of metabodeconplus. The CluPA algorithm was chosen because it offers reliable alignment between target and reference spectra and has been implemented in R, which facilitated its addition to the metabodeconplus R package. Furthermore, the alignment approach has been extended by the new snap_to_ref function to remove the residual positional uncertainty that remains after CluPA.

In addition, the underlying deconvolution algorithm was optimized for speed and accuracy.

The contribution of metabodeconplus is thus a single, automated end-to-end workflow for 1D NMR fingerprinting: from raw spectra, it produces a two-dimensional table of aligned signal integrals that feeds directly into built-in sample classification. It achieves this by integrating three previously separate steps (deconvolution, signal alignment via the adapted CluPA algorithm, and classification) into one package and by automating the parameter search of these steps. A substantially faster deconvolution backend allows the application to large datasets containing several hundred spectra. For a user, this means the entire path from raw spectra to a classification-ready feature matrix is handled by one package, replacing the ad-hoc combination of separate deconvolution and alignment tools that this analysis previously required.

## 2. Materials and Methods

### 2.1. Package Availability

The R package is available on GitHub at https://github.com/spang-lab/metabodeconplus and on CRAN at https://cran.r-project.org/package=metabodeconplus. Both were accessed on 11 August 2026. All results reported here were produced with metabodeconplus versions 0.20.0 to 0.22.2 under R 4.5.3, using the R packages ranger 0.18.0 and speaq 2.7.0, and the Rust backend mdrb 0.0.5, built with Rust 1.94.0.

### 2.2. Study Cohorts

Four datasets were used throughout this study. The Urine dataset is distributed with the package, and the Sim3, Blood and AKI datasets can be obtained via download_example_datasets() or downloaded manually from GitHub.

The Sim3 dataset consists of 100 simulated spectra, each containing 2048 data points covering a chemical shift range of 3.59 to 3.28 ppm (datapoint spacing 0.00015 ppm). The spectra are evenly split into two groups, A and B (50 each). Each spectrum contains the same 25 underlying Lorentzian signals: a fixed set of reference signal positions, signal intensities, and half-widths is reused across all spectra, with per-spectrum variability introduced by (i) a scalar global ppm shift drawn from $N(0,0.00120^{2})$ ppm (SD =8 datapoints), (ii) a per-signal ppm jitter drawn from $N(0,0.00060^{2})$ ppm (SD =4 datapoints), (iii) per-signal amplitude scaling drawn from U[0.4,1.6], (iv) per-signal half-width scaling drawn from U[0.9,1.1], and (v) additive Gaussian noise. In group A, the amplitudes of 3 of the 25 signals (the discriminating signals) are additionally scaled by fixed factors of 1.35, 1.35, and 1/1.35 (≈0.74); group B is left unmodified. The first spectrum is generated without any positional shift and serves as a clean unshifted reference. The shared reference parameters were derived from an initial deconvolution of the Blood dataset with manually optimized settings so that the simulated spectra closely resemble real blood plasma spectra while providing exact ground truth for signal parameters, group labels, and noise levels.

The Blood dataset consists of 16 human blood plasma spectra measured with a 1D ^1^H Carr–Purcell–Meiboom–Gill (CPMG) pulse sequence.

The Urine dataset consists of 2 human urine spectra measured with a 1D ^1^H NOESY pulse sequence. Specimens from both the Blood and Urine data sets were obtained from the German Chronic Kidney Disease (GCKD) study collected at the baseline time point [23,24]. The GCKD study was carried out in accordance with the Declaration of Helsinki, registered in the German Register of Clinical Trials (DRKS 00003971), and approved by the ethics committees of the participating institutions.

The AKI dataset consists of 106 urinary 1D ^1^H NMR spectra acquired with a 1D ^1^H NOESY pulse sequence in the context of a study on acute kidney injury (AKI) following cardiac surgery with cardiopulmonary bypass (CPB) use [25]. Urinary specimens were collected 24 h after surgery. These spectra had been previously collected with written informed consent of the patients upon ethical approval from the University Clinic Erlangen, Erlangen, Germany. The study was approved by an institutional review board at the Faculty of Medicine at the Friedrich-Alexander University (FAU) Erlangen-Nuremberg under the identifier #4010 [26].

All experimental measurements for these datasets were performed on a 600 MHz Bruker Avance III spectrometer (Bruker BioSpin GmbH, Rheinstetten, Germany) employing a helium cooled cryogenic probe and a cooled sample changer. The measurements were performed using optimized pulse sequences for the suppression of water signals.

An overview of all datasets is given in Table 2.

### 2.3. The Deconvolution Method

**Figure 1:**
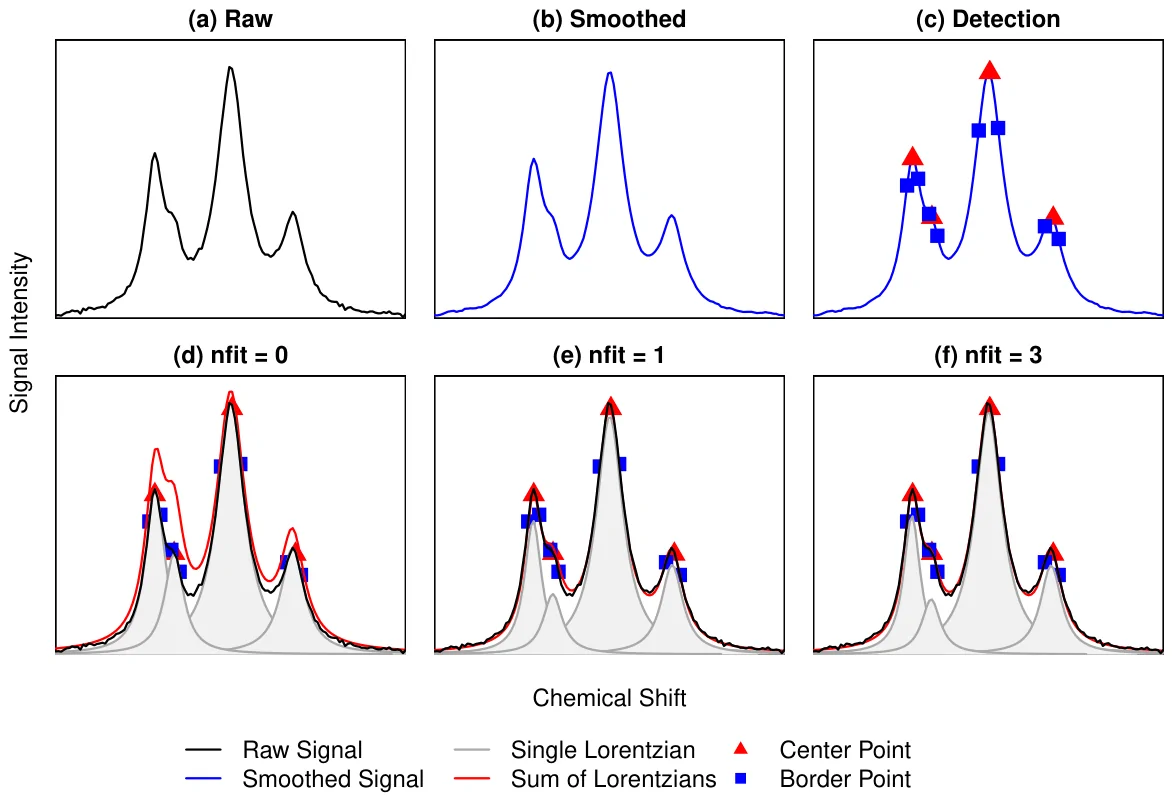
Visualization of the deconvolution algorithm for a small, simulated spectrum with four peaks. (**a**) Raw spectrum. (**b**) Smoothed spectrum after applying a moving average filter. (**c**) Peak detection on the smoothed spectrum: red triangles mark peak centers, blue squares mark peak borders. (**d**–**f**) Lorentzian curves after nfit = 0, 1 and 3 iterations of refinement. In (**d**–**f**), the black line shows the raw spectrum and the red line shows the superposition of the fitted Lorentzian curves.

The input for metabodeconplus is a set of Fourier-transformed, phase- and baseline-corrected, and properly referenced 1D ^1^H NMR spectra. The deconvolution algorithm used in metabodeconplus was first described in Koh et al. (2009) [27] and implemented as part of MetaboDecon1D v0.2.2 [14]. The implementation in metabodeconplus was completely restructured into a modular framework with four distinct stages: (i) smoothing, (ii) peak detection, (iii) peak filtering, and (iv) Lorentzian function fitting. Throughout, signal refers to a resonance or other contribution that raises the measured intensity at a given position, and each detected signal is approximated by one Lorentzian curve, whose maximum is called its peak. A concise overview of the major functional differences between MetaboDecon1D v0.2.2 and metabodeconplus is provided in Table A1. The overall algorithm is visualized for a small, simulated spectrum in Figure 1.

#### 2.3.1. Smoothing

Smoothing is required to suppress spurious detections caused by noise. Following Koh et al. (2009) [27], a moving average filter is applied to the spectral intensities before peak detection. A moving average filter is a low-pass filter that replaces each intensity value with the equally weighted average of the values within a surrounding window of user-defined size *m*. Denoting the spectral intensities as $Y_{\left(0\right)}=Y$, the chemical shift at data point *i* as $\omega_{i}$, the half window $h=\lceil \frac{m}{2}\rceil$, and the number of points *N*, one pass of the filter is given by

(1) $$Y_{\left(k\right)}\left(\omega_{i}\right)=\frac{1}{m}\sum_{j=1}^{m}Y_{\left(k-1\right)}\left(\omega_{i-h+j}\right),\;i\in \left[h,N-h\right].$$

It is applied iteratively *n* times, yielding the smoothed spectral intensities $Y_{\left(n\right)}=Z$.

#### 2.3.2. Peak Detection

Peak detection operates on the smoothed spectral intensities using the curvature-based method introduced by Koh et al. (2009) [27]. Peaks are identified based on local minima in the second derivative of the smoothed spectrum. Each peak is represented by a left, center, and right point $k_{i}\in \left\{1,\ldots ,N\right\}$, with i=1,2,3, respectively. Each peak is assigned the score introduced by Koh et al. (2009) [27]:

(2) $$\mathrm{score}=\min\left(\sum_{i=k_{1}}^{k_{2}}|Z^{\prime \prime }\left(\omega_{i}\right)|,\;\sum_{i=k_{2}}^{k_{3}}\left|Z^{\prime \prime }\left(\omega_{i}\right)\right|\right),$$

i.e., the smaller of the two cumulative absolute second-derivative sums over the peak’s left and right halves. Sharper, more prominent peaks therefore receive higher scores.

#### 2.3.3. Peak Filtering

Peaks in the signal-free region are used to estimate the noise distribution. Only peaks with a score exceeding μ+δ·σ are retained, where μ and σ are the mean and standard deviation of the noise scores and δ is a user-tunable multiplier (default 6.4) that controls peak-detection sensitivity. Peaks inside user-specified ignore regions, such as the water-artifact region, are also removed.

#### 2.3.4. Lorentzian Function Fitting

Each detected peak is characterized by a triplet of observed points $\left(\omega_{i},y_{i}\right)$, i=1,2,3, which uniquely determines the center $\omega_{0}$, half-width λ, and amplitude *A* of a Lorentzian function passing exactly through those three points. These are the initial parameter estimates. When the resulting Lorentzian curves are superimposed, the sum tends to exceed the observed spectrum due to spill-over from neighboring peaks. Following Koh et al. [27], each peak’s height is, therefore, iteratively reduced by a rule of proportion until the superposition matches the raw spectrum. Compared to the original MetaboDecon1D implementation, metabodeconplus uses a more efficient strategy for computing the Lorentzian parameters during both initial estimation and iterative refinement; mathematical details are provided in Appendix C.

#### 2.3.5. Rust Implementation

In addition to the native R implementation, metabodeconplus provides an optional Rust backend that implements the same deconvolution algorithm with further refinements for numerical stability and computational efficiency. The backend is accessible through the standard R interface via the use_rust parameter of deconvolute() and yields results that are numerically very close, though not identical, to the R implementation. The substantially reduced runtimes allow for a systematic grid search of optimal deconvolution parameters (Section 2.5.1) even for large datasets consisting of several hundred spectra.

#### 2.3.6. Scoring of Deconvolution Quality

To score the deconvolution quality of simulated data or controlled measurements of known compounds, a new measure called PRARPX was devised. It scores both the number of correctly identified Lorentzians and the approximation of the measured spectra. The Extended Peak Ratio compares the correctly identified Lorentzians *c* to the true Lorentzians *t* plus the incorrectly identified Lorentzians *i*,

(3) $$\mathrm{PRX}=\frac{c}{t+i},$$

and the Area Ratio is the total absolute residual *R* over the total absolute observed signal *S*,

(4) $$\mathrm{AR}=\frac{R}{S}.$$

PRARPX is the product of the two,

(5) PRARPX=PRX·max(0,1−AR).

Both factors fall within [0,1]. The score approaches 1 the more unique true Lorentzians are assigned a fitted Lorentzian and the smaller the residual becomes. Therefore, PRARPX approaches 1 for an ideal deconvolution. Because PRARPX requires ground-truth Lorentzian parameters, it is not applicable to biological specimens containing unknown compound numbers. Appendix D motivates it in detail and contrasts it with using PR or AR alone.

### 2.4. The Alignment Method

**Figure 2:**
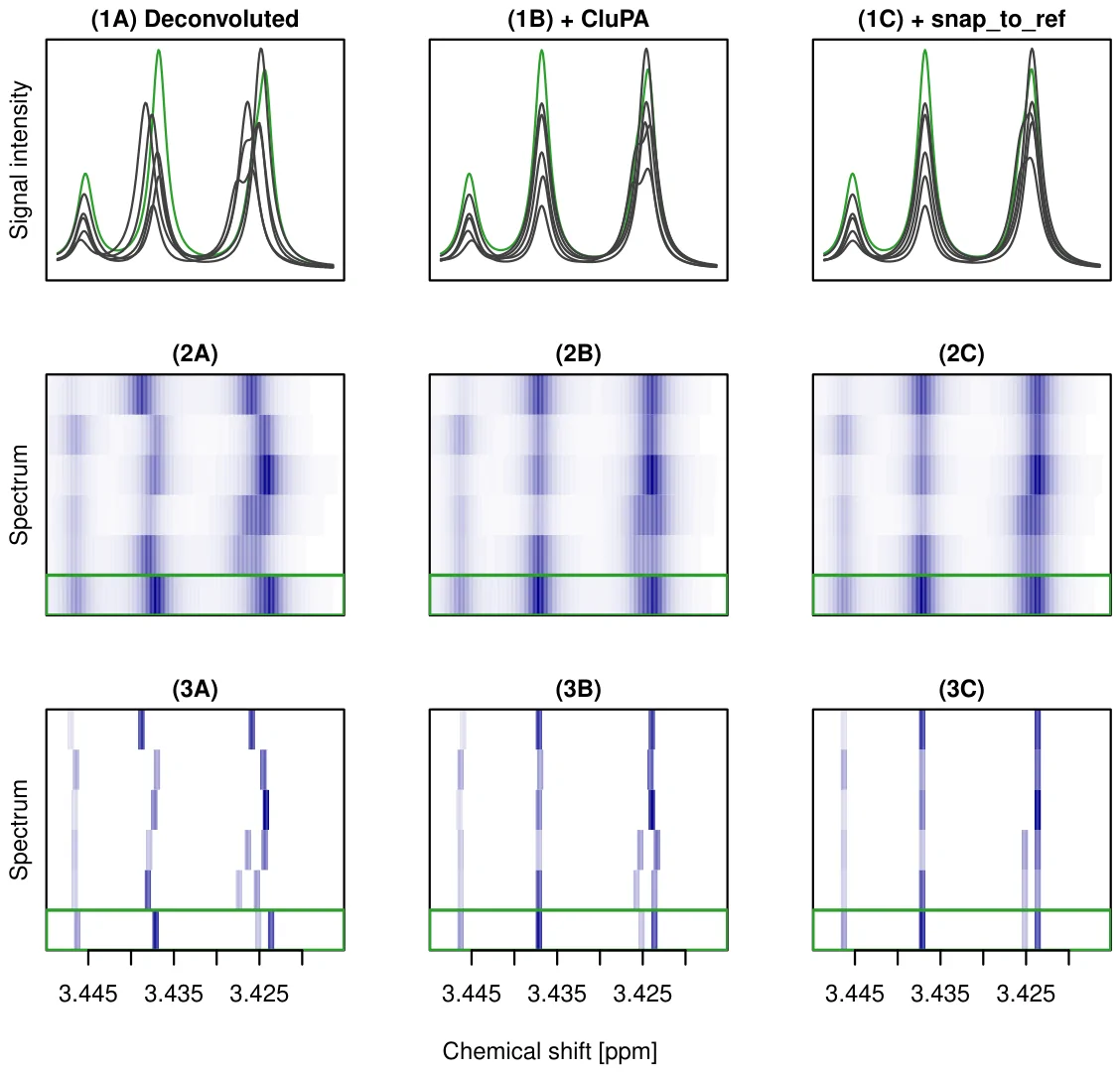
Cumulative effect of the two-step alignment pipeline on the first six Sim3 spectra over 3.415–3.450 ppm, aligned with maxShift = 4 and maxCombine = 5. Columns are the stages: after deconvolution (**1A**–**3A**), after CluPA (**1B**–**3B**), after snap_to_ref() (**1C**–**3C**). Top row (**1A**–**1C**): overlay of the reconstructed spectra. Middle row (**2A**–**2C**): the same spectra as intensity heatmaps, where darker is higher. Bottom row (**3A**–**3C**): one vertical dash per fitted Lorentzian, at its center, shaded by its integral. The reference spectrum is green throughout.

Proper signal alignment is crucial to correct for chemical-shift variations across samples and to ensure that subsequent statistical analyses compare the same signals from the same metabolites. Following deconvolution, the obtained peak lists are passed through two consecutive steps: a coarse CluPA (cluster-based peak alignment) shift, followed by a refinement step snap_to_ref(). Both operate on the discrete peak grid: CluPA shifts whole spectrum segments by the same integer-datapoint offset, so all peaks within one segment move together. One peak may end up well-aligned while another in the same segment is still slightly off. The refinement step removes this residual mismatch by snapping each remaining peak individually onto the nearest peak column of the reference spectrum. The two steps are exposed individually as clupa() and snap_to_ref() and are chained by the wrapper align(). The combined procedure is visualized for a set of simulated spectra in Figure 2.

#### 2.4.1. CluPA

The first stage is based on the hierarchical cluster-based peak alignment (CluPA) approach from the speaq package [11,22]. CluPA aligns a target spectrum to a reference spectrum in two phases. First, it shifts the spectrum globally. Then, it iteratively splits the spectrum into smaller local segments that are individually aligned. The segment boundaries are defined by hierarchical clustering applied to the combined peak list of the reference and target spectrum: peaks are grouped into a tree based on their distances, and at each iteration, all segments are bisected according to the dendrogram. For each segment, the optimal integer datapoint shift is determined by fast Fourier transform cross-correlation, bounded by the user-supplied tolerance maxShift. Iterations stop when a segment contains only peaks from the same spectrum or fewer than three peaks. CluPA changes peak centers but does not change the number of peaks per spectrum.

#### 2.4.2. snap_to_ref

CluPA shifts whole spectrum segments, not individual peaks, so peaks within the same segment all move equally in terms of direction and distance. Following CluPA, the alignment of spectra is substantially improved, but individual peaks are still not guaranteed to exhibit the exact same chemical shift across spectra, and a refinement step is needed to enforce a common peak grid. The snap_to_ref() step does this by collapsing (snapping) each spectrum’s peak list onto the peak grid of the reference spectrum chosen by CluPA. For each peak, the nearest reference peak within maxCombine columns is its target. Peaks farther than maxCombine from every reference peak are dropped. If multiple peaks are snapped to the same reference, their integrals are summed when the feature matrix is built. Rare signals not present in the reference spectrum may be lost or attributed to the wrong reference. The interpretation of maxCombine is, therefore, the residual positional uncertainty that remains after CluPA. Figure 2 visualizes the cumulative effect of the two steps: the top row overlays the six reconstructed spectra after each step, the middle row renders them as intensity heatmaps, and the bottom row reduces each spectrum to its sparse peak-position marks.

Both clupa() and snap_to_ref() align/snap each spectrum towards a fixed reference spectrum. This makes application to new samples trivial: each new sample is aligned and snapped to the same reference.

### 2.5. Parameter Optimization

#### 2.5.1. Unsupervised Parameter Optimization

Metabodeconplus’s deconvolution exposes four tunable parameters: nfit, smit, smws and delta. nfit sets the number of iterative Lorentzian-refinement passes, smit the number of moving-average smoothing passes, smws the smoothing window size (in datapoints), and delta the noise-threshold multiplier from Section 2.3.3. Without class labels, these can be optimized for each spectrum individually in an automated fashion by minimizing the Area Ratio (AR) between observed intensities *Y* and reconstructed intensities $\hat{Y}$:

(6) $$\mathrm{AR}=\frac{\sum_{i}\left|Y\left(\omega_{i}\right)-\hat{Y}\left(\omega_{i}\right)\right|}{\sum_{i}\left|Y\left(\omega_{i}\right)\right|},\;i\in \left\{1,\ldots ,N\right\}.$$

For evenly sampled spectra, this is the mean absolute residual divided by the mean absolute signal, and smaller values indicate a better fit.

Minimizing the AR alone is not always optimal as the AR keeps decreasing as more Lorentzians are fitted, so the optimum may drift towards over-fit reconstructions with many spurious peaks. Therefore, the maximum number of expected signals npmax was added as a user-defined parameter. The automated parameter optimization of the deconvolution step then sweeps a 60-cell parameter grid smit∈{1,2,3}, smws∈{3,5,7,9}, delta∈{1.6,3.2,4.8,6.4,8.0}, nfit=10 and picks the smallest-AR cell whose peak count does not exceed npmax. As a consequence, the user has to set only npmax manually. A suitable npmax can be set from domain knowledge (e.g., expected maximum signal numbers in blood or urine spectra) or chosen by maximizing downstream classification performance in cases where class labels are available (Section 2.5.2).

The 60-cell grid above was chosen by deconvoluting representative blood and urine spectra over a broader candidate grid, visually inspecting the resulting Lorentzian reconstructions, and trimming the grid to the values that consistently produced plausible fits. The delta step of 1.6 was picked so that the previous hand-tuned default delta=6.4 falls on a grid point. Users who want finer control can supply their own parameter grid.

When neither domain knowledge nor class labels are available, passing npmax=−1 derives the value from the data as the elbow of the curve relating AR to the number of fitted signals, located with the Kneedle heuristic [28] (Appendix G).

#### 2.5.2. Supervised Parameter Optimization

When class labels are available, npmax, maxShift and maxCombine can be optimized jointly by maximizing classification performance on the resulting feature matrix. Because the same metabolites may not be detectable in all spectra, zeros in the feature matrix encode “missing signal” rather than “zero area”. Linear models confound these zeros with small observed values and become unstable. Therefore, a non-linear random forest classifier from the ranger package [29] was used. It can partition based on the presence of a signal as well as based on its magnitude. Random forests also performed well in a systematic comparison of classification algorithms on metabolic ^1^H NMR fingerprints [30]. A further advantage of random forests is the out-of-bag (OOB) error [31], a generalization-error estimate obtained during training itself. The OOB error does not require an extra held-out split or inner cross-validation loop. The two alignment parameters, maxShift (CluPA tolerance) and maxCombine (snap-to-ref window width), are governed by different sources of positional error, and their empirical optima do not necessarily coincide, so tuning them jointly is in general not redundant. All three parameters can therefore in principle be swept as a three-dimensional grid via the supervised search in fit_mdm().

In practice, tuning all three at once is usually not recommended, as the runtime grows multiplicatively with the size of each axis, and the deconvolution and CluPA stages dominate that cost. Instead, the following two-step procedure is recommended:

1. Pick npmax from domain knowledge (e.g., urine spectra typically contain more metabolites than blood), by visual inspection of a representative deconvolution (Section 2.5.1), or by setting npmax=−1 to leave it to the elbow heuristic [28] of Appendix G.
2. Rely on CluPA’s internal optimum by setting maxShift=−1. This triggers an adaptive sweep that doubles maxShift through {1,2,4,8,…}, runs CluPA at each step, computes the average pairwise Pearson correlation of the aligned Lorentzian superpositions, and stops one step before that correlation first decreases.

The supervised search is then reduced to a one-dimensional sweep over maxCombine, which is by far the cheapest of the three stages to rerun.

fit_mdm() relies on caching and multiprocessing to keep its runtime reasonable. Three optimizations are combined:

1. Each npmax value has an associated set of per-spectrum deconvolution parameters that give the lowest reconstruction error. Finding them requires an internal grid search per spectrum, which is run once at function entry and attached to each spectrum. Whenever npmax changes, the optimal parameters can be looked up instead of repeatedly recomputed.
2. The grid is traversed with npmax varying slowest, then maxShift, then maxCombine. If npmax is unchanged between two rows, the deconvolution of the previous row is reused instead of recomputed; if npmax and maxShift are both unchanged, the CluPA alignment is reused as well.
3. Deconvolution, alignment, and fitting use several workers each. Parallelization is done with respect to spectra and not with respect to grid rows to keep memory low. Parallelization over grid rows would force every worker to hold a copy of all spectra, whereas for parallelization over spectra, each worker only holds the spectra it is currently processing.

## 3. Results

### 3.1. Deconvolution Quality on Sim3: Metabodeconplus vs. MetaboDecon1D and Grid Search

Deconvolution quality was scored with PRARPX as defined in the materials and methods section. To this end, a simulated dataset Sim3 (see Table 2 for details) was used. Note that this simulated dataset also contained noise to approximate a realistic experimental data set. Each of the 100 Sim3 spectra was deconvoluted with MetaboDecon1D (default parameters), metabodeconplus (default parameters), and metabodeconplus with unsupervised grid-search parameter selection at npmax∈{10,15,20,25,30,35,40}. The signal-free region was auto-derived per spectrum so that all methods saw equivalent inputs. Note that PRARPX is computed from ground-truth Lorentzian parameters, whereas the general grid search parameter optimization has to operate on the Area Ratio alone.

**Figure 3:**
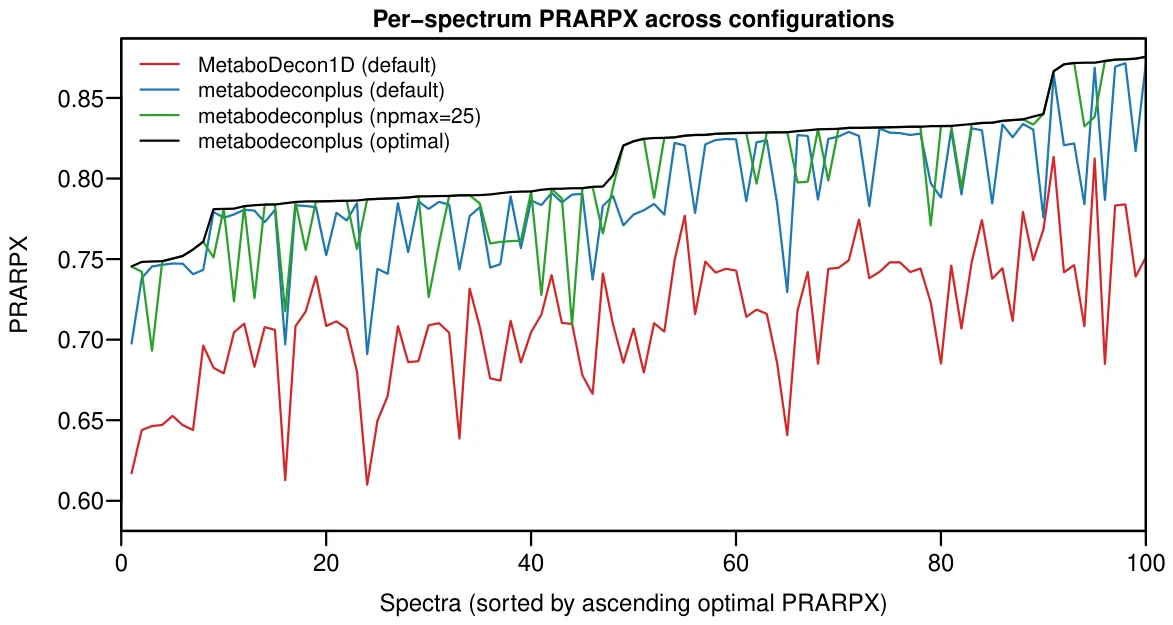
Per-spectrum PRARPX on the 100 Sim3 spectra for the configurations of Table 3: MetaboDecon1D default (red), metabodeconplus default (blue), the best-mean grid-search configuration (green), and the per-spectrum optimum, i.e., the best parameter combination for each individual spectrum (black). Spectra are sorted by ascending per-spectrum optimum so that the *x*-axis runs from the hardest spectrum on the left to the easiest on the right.

Table 3 summarizes PRARPX (mean, SD, min, max) across all spectra for each configuration, and Figure 3 plots the per-spectrum trace. The default metabodeconplus configuration already improved on MetaboDecon1D, and the grid-search cutoff at moderate npmax closed part of the remaining gap to the metabodeconplus optimal bound. The gap is smallest at npmax=25; smaller and larger budgets are both worse, and each settles onto a plateau (Table 3).

**Figure A2:**
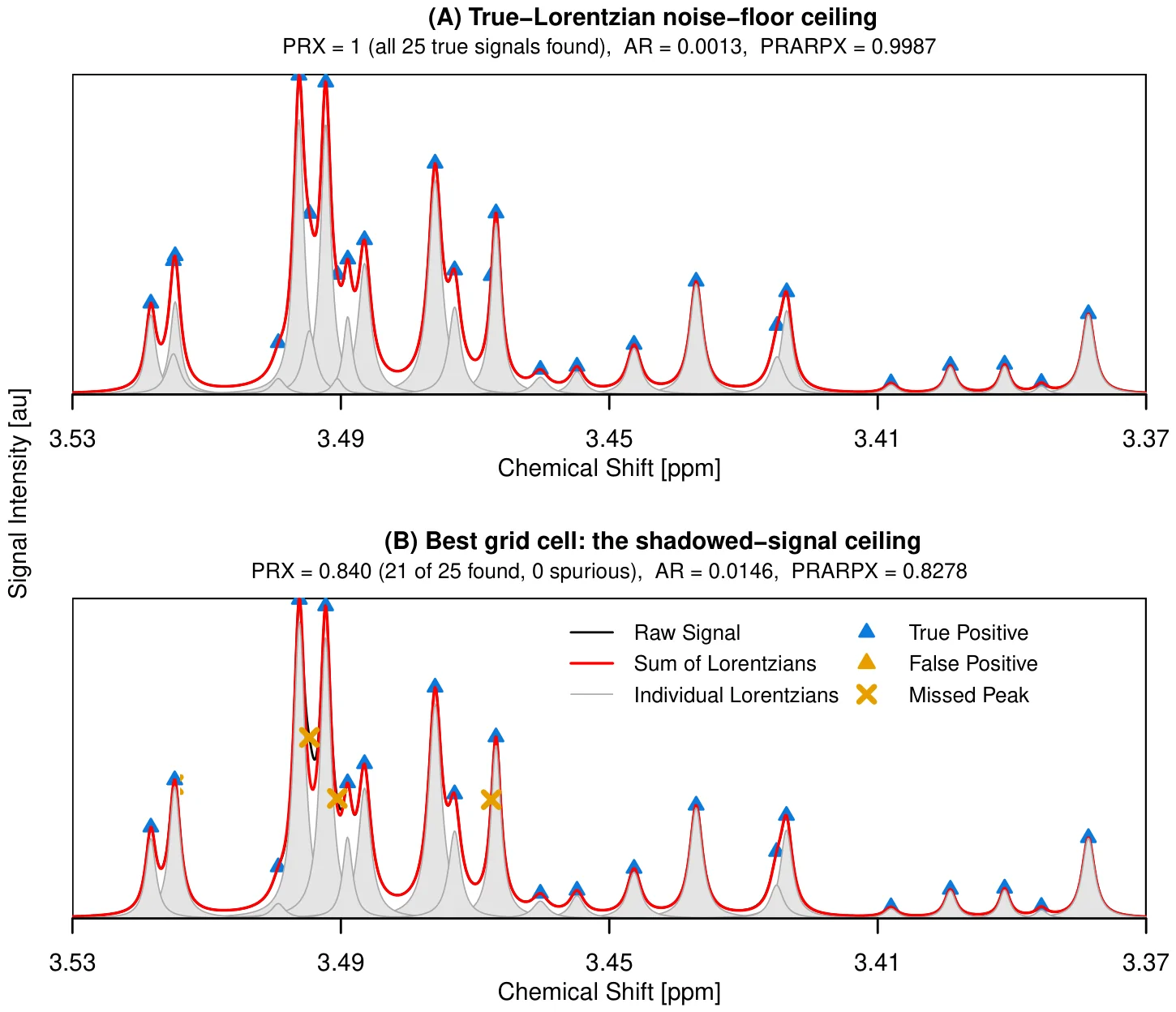
Two intrinsic upper bounds on PRARPX, illustrated on the zero-shift reference spectrum of the Sim3 dataset. (**A**) The true-Lorentzian superposition (red) overlaid on the observed intensities (black), with the individual true Lorentzians shown as gray fills. PRX =1 by construction (every true signal is present); the residual area equals the additive measurement noise and yields AR>0, so even this noiseless reconstruction cannot reach PRARPX=1. (**B**) The deconvolution at the best-PRARPX combination of metabodeconplus’s default grid of 60 combinations. Signals shadowed by larger neighbors produce no detectable curvature minimum and are therefore not recovered, pulling PRX below 1. Together, the two panels visualize the noise-floor and shadowed-signal ceilings discussed in Section 3.1.

Even with the metabodeconplus optimal parameters, an ideal value of PRARPX=1 is not reached for two reasons. First, the superposition of the estimated noiseless Lorentzian signals cannot fully reproduce the noisy observation. Figure A2A illustrates this. Second, small signals completely shadowed by larger neighbors produce no detectable curvature minimum and are therefore not recovered, so PRX<1 for ordinary spectra (Figure A2B).

### 3.2. Alignment Quality on Sim3: CluPA and snap_to_ref

Every Sim3 spectrum contains the same 25 simulated signals named A–Y at known positions, and the first spectrum was simulated without any per-spectrum jitter or global shift, so it serves as the unshifted reference. This allows alignment quality to be quantified directly. For each extracted peak, it was recorded which of these 25 simulated signals it corresponded to: a peak whose fitted center lay within ±2 data points of a simulated position inherited that signal’s name; a peak farther away from any simulated position was labeled Z (a spurious detection). Labels are assigned to the raw deconvolution output, before any alignment. Therefore, each peak carries the identity of the simulated source it came from through the rest of the pipeline.

Deconvolution with default parameters returns 18–22 peaks per Sim3 spectrum over the 0.15 ppm interval holding its 25 simulated signals. On 100 windows of that width drawn at random from the analyzed regions of the urinary AKI spectra, it returns 0–47 (median 23), so the average simulated signal density is typical of real experimental spectra, although this also shows that real experimental spectra contain especially challenging regions with a very high signal density.

**Figure 4:**
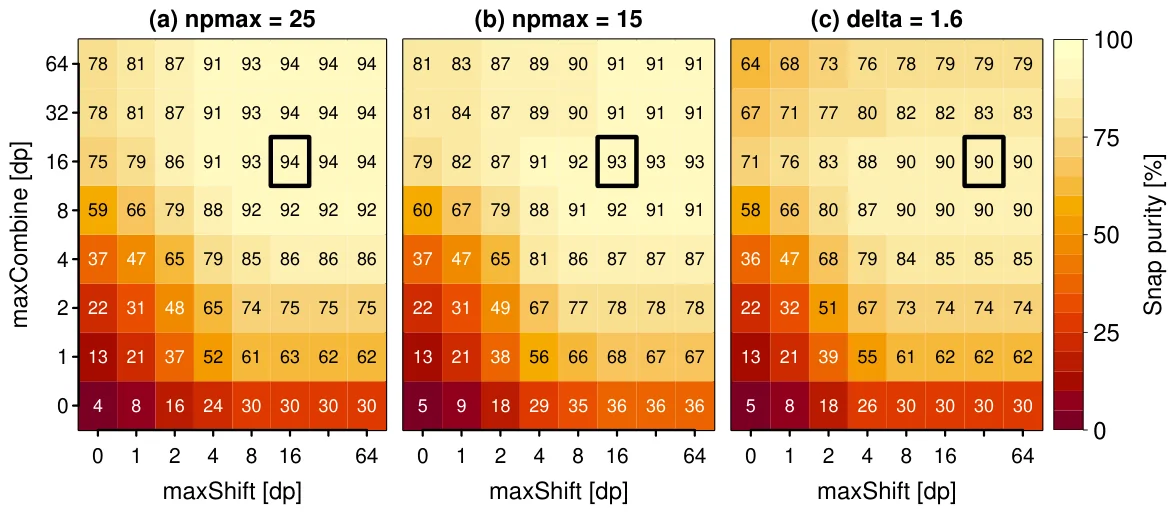
Snap purity over the maxShift×maxCombine grid at three deconvolution settings: (**a**) npmax=25, (**b**) npmax=15, (**c**) smit=1, smws=3, delta=1.6, nfit=10. Purity is defined in Section 3.2 and is calculated in each panel over the reference signals that panel recovered, 21, 18 and 21 of 25. In (**a**), neither step suffices alone: 30% with CluPA alone (bottom row) and 78% with snapping alone (left column), against 94% combined. Note that for clarity, no decimals are shown. Purity saturates past maxShift=16. The outlined square highlights the cell with maximum purity.

CluPA moves whole spectrum segments by a common integer offset, so peaks may not be matched exactly. snap_to_ref() then moves each peak to the nearest peak column of the unshifted reference spectrum, dropping peaks with no such column within maxCombine datapoints. A signal counts as recovered when the reference spectrum carries a fitted peak within two datapoints of its simulated position, and only recovered signals have a reference column, so how many columns exist is a property of the deconvolution and is stated per panel. A (spectrum, signal) pair counts as correct when its reference column receives that signal’s peak and no other. The fraction of correct pairs over the 100 spectra and the reference columns of the panel is the snap purity reported in Figure 4.

Results show that the snap purity is highest where CluPA has already brought peaks within a small maxCombine window of their reference columns. Widening the window past that point costs essentially nothing only when the deconvolution recovers every reference signal and fits no spurious Lorentzians so that no peak is available to be mis-snapped: at npmax=25, purity is flat at 94% out to maxCombine=64 (0.0 points, Figure 4a). If either condition fails, i.e., signals are missed (npmax=15, Figure 4b) or spurious Lorentzians are fitted, due to a modified noise threshold (delta=1.6, Figure 4c), peaks are swept onto wrong reference neighbors and a clear valley appears, costing 2.0 and 11.5 points by maxCombine=64; it is deepest in (c), where on average, 30.5 peaks are fitted per spectrum against 25 simulated ones, so correspondingly more peaks can be swept into an occupied column.

### 3.3. Supervised Parameter Optimization on Sim3

In the following, it was investigated whether the supervised classification-based search described in Section 2.5.2, where class labels are known, is feasible. To this end, a 5000-tree ranger probability forest was run over a small grid. The grid spans all three parameters (npmax∈{10,15,20,25,30,35,40}, maxShift∈{2,4,8,16,32}, maxCombine∈{1,5,10}). This Sim3 sweep is the only instance where all three parameters were tuned at once. The small spectrum count and coarse grid keep the runtime under a few minutes. This made it possible to analyze whether the supervised optimum of parameters obtained here is consistent with the unsupervised one. The grid search selected npmax=20, maxShift=16, maxCombine=5 as the best combination (OOB accuracy 70%, OOB AUC 68.8%). Applied to the held-out 50 test spectra, this configuration achieved a test accuracy of 68% and AUC of 80.0%. Of the top-10 features ranked by ranger permutation importance, 3 fall within 3 datapoints of a discriminative signal.

The supervised optimum npmax=20 is close to, but not identical with, the unsupervised optimum npmax=25 of Section 3.1. The supervised search optimizes classification accuracy, the unsupervised one reconstruction fidelity, so the two criteria need not select the same value. In contexts where signal recovery rather than classification accuracy is the criterion of interest, npmax is more naturally set to a prior upper bound on the expected signal count than tuned by the supervised search.

**Figure 5:**
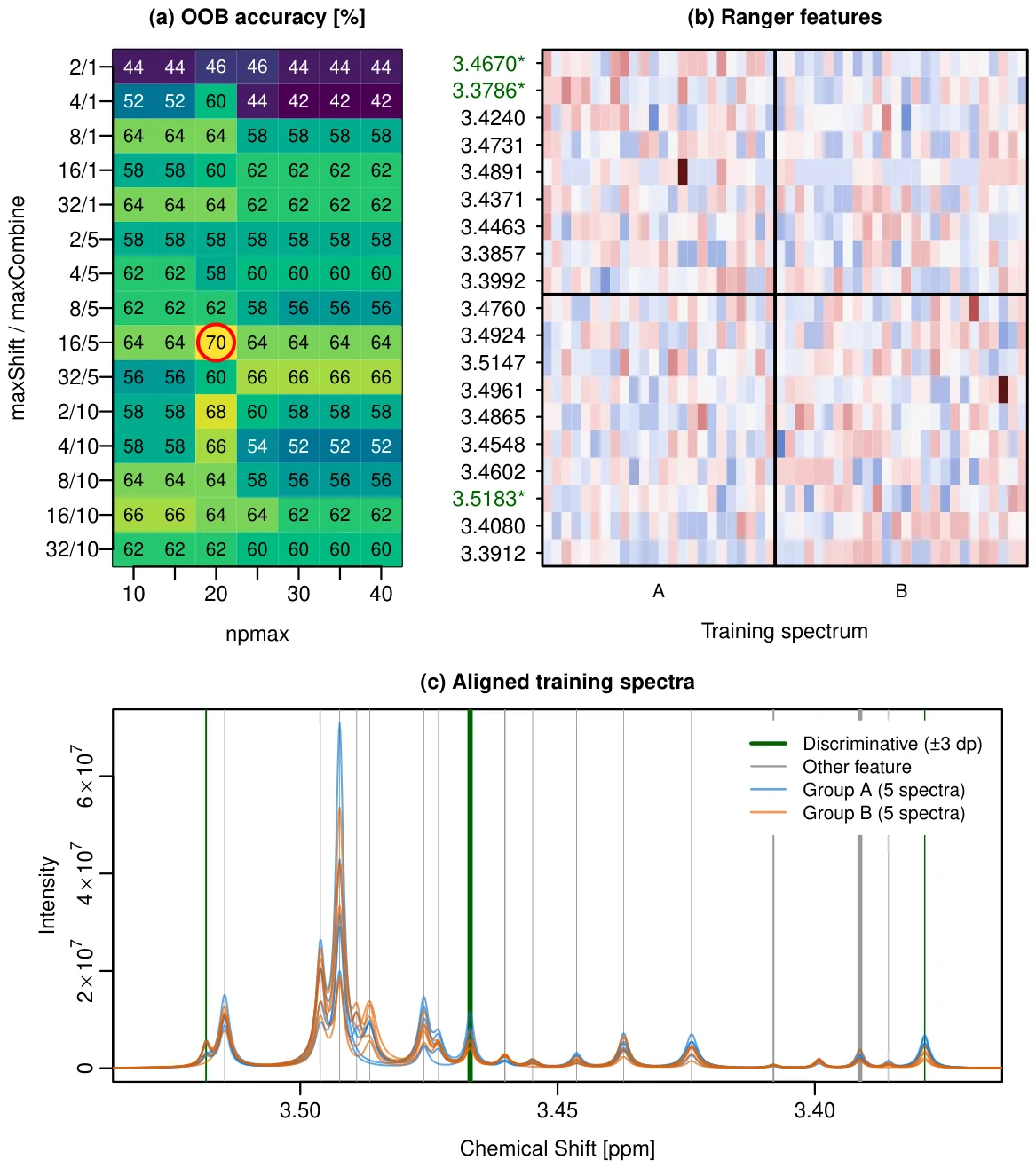
Supervised parameter optimization on Sim3. (**a**) Out-of-bag accuracy for every (maxShift,maxCombine) row × npmax column combination; rows are grouped by maxCombine and sorted by maxShift ascending within each group. The combination with the highest OOB accuracy, ties broken by AUC, is circled in red. (**b**) Heatmap of all ranger features obtained for the best parameter combination. Rows above the divider are features whose mean is higher in Group A (positive two-sample t-score); rows below are higher in Group B. Within each half, rows are sorted by ranger permutation importance. Each row is scaled to zero mean and unit variance across spectra, so color shows relative, not absolute, intensity. Tick labels of features within ±3 datapoints of a discriminative signal are colored dark green and marked with an asterisk. Columns are training spectra, labeled by class (A, B). (**c**) Superposition of 5 group-A and 5 group-B training spectra after alignment and snapping over the 3.53–3.37 ppm window, with one vertical line per feature shown in (**b**). Green lines mark features within ±3 datapoints of a discriminative signal; gray lines mark all other ranger features. Line width scales with the ranger permutation importance.

Data show that automated parameter optimization selects a working configuration, reaching an out-of-bag accuracy of 70%. As can be seen from Figure 5a, classification performance depends strongly on the parameter settings: only 1 of the 105 combinations attains that value. Figure 5b shows that the true discriminatory features are among the most informative features picked by the classification algorithm [29,31]. The fact that other features are also considered by the classification algorithm is due to the fact that the three discriminatory features of group A were up- and downscaled only very moderately. Furthermore, a per-signal amplitude scaling and the addition of Gaussian noise were performed on the Sim3 data set as described in the Study cohorts section. As can be seen from Figure 5c, the three discriminatory signals are of only moderate intensity and show partial overlap with other signals. Therefore, the Sim3 dataset provides a challenging realistic example.

### 3.4. End-to-End Prediction Performance on the AKI Dataset

The end-to-end predictive performance was evaluated on the AKI dataset (34 AKI versus 72 control urinary spectra, creatinine-normalized) by comparing three models. The first was an equidistant-binning baseline that mirrors the fixed-bin feature representation originally used by Zacharias et al. [25], who introduced the AKI dataset. To this end, a 700-bin feature matrix consisting of 300 bins covering 6.5–9.5 ppm and 400 bins covering 0.5–4.5 ppm (at a fixed bin width of 0.01 ppm) was used. Zacharias et al. [25] employed a support-vector machine (SVM) for classification. For the current contribution, the SVM was replaced with a ranger-based random forest [29,31,32] run in probability mode (one class probability per sample). This matched the classifier used by the metabodeconplus pipeline and ensured a fair comparison.

The second was the full metabodeconplus pipeline: deconvolute → CluPA → snap_to_ref → ranger, with npmax=−1. The third was speaq 2.0 [11], the only tool of Table 1 that extracts features without a metabolite reference library and runs unattended from R, so that it can be driven from the same cross-validation harness. Its wavelet based peak detection, peak grouping and feature-matrix construction were run on the same 0.5–4.5 and 6.5–9.5 ppm windows at package defaults, except for the baseline threshold, which was lowered from 1000 to 100 because it is an absolute intensity cut-off and the creatinine-normalized AKI spectra are about three orders of magnitude smaller than the data speaq’s default is calibrated for. This yielded 426 cross-sample peak groups, classified with the same ranger setup on the same folds.

**Figure A5:**
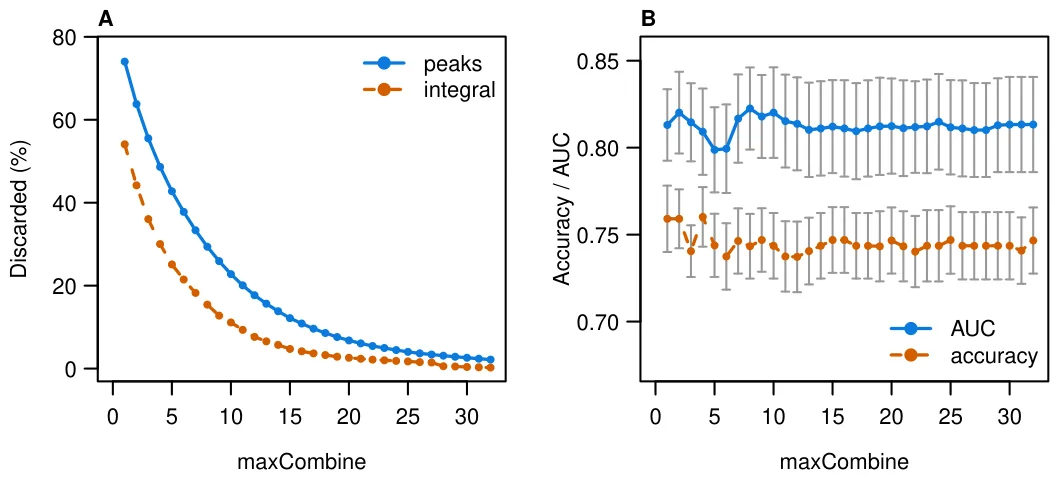
Cost of snap_to_ref() on the AKI cohort at each maxCombine, over the 106 urinary AKI spectra and 111,494 fitted Lorentzians as described in Section 3.4 (npmax=1117, maxShift=8). (**A**) Percentage of fitted Lorentzians with no reference column within maxCombine, and their share of the fitted integral. (**B**) Accuracy and AUC of the protocol of Section 3.4 on the resulting feature matrix, mean ± one standard error over the 30 folds; deconvolution, alignment and snapping are run once over the cohort and the feature matrix is subset per fold, as for the speaq 2.0 baseline.

For metabodeconplus, npmax was left to the elbow heuristic of Appendix G by setting it to −1, which resolves to an npmax of 1117 on the full AKI dataset and to 1105–1130 across the 30 training folds. The test further relies on CluPA’s internal optimum for maxShift, again by setting it to −1 (see Section 2.5.2), so that only maxCombine is swept on the training fold of each cross-validation split via the inner grid-search in fit_mdm(). At the maxCombine selected on the full AKI dataset for optimal classification performance (maxCombine=2), snapping discards 63.8% of the 111,494 Lorentzians fitted on all spectra of the dataset and merges 0.1%; across maxCombine from 1 to 32, the discard rate runs from 74.0% to 2.2% while accuracy and AUC stay flat (Figure A5).

Predictive performance was estimated by stratified 10-fold cross-validation repeated over three random seeds (30 folds in total per model). Each fold’s training portion was independently re-fit before scoring on the held-out fold, and standard errors are taken across folds. The binning baseline reached 75.4 ± 2.33% accuracy and AUC = 0.810 ± 0.027; metabodeconplus reached 73.7 ± 2.20% and AUC = 0.827 ± 0.025, thus matching the binning baseline with no significant loss or gain in accuracy or AUC, while operating on a higher-resolution feature matrix whose columns correspond to individually localized signals rather than fixed-width bins. Paired over the 30 shared folds, none of the three representations differs significantly from another in terms of accuracy or AUC (smallest Holm-adjusted p=0.69 [33], Table A3).

On the same folds, speaq 2.0 reached 76.6 ± 2.01% and AUC = 0.814 ± 0.024. The figures obtained by speaq 2.0 are not directly comparable with those of the other two approaches, which already differ in what a feature measures: a bin sum, a wavelet peak height, and a fitted line-shape integral. The peak grouping is a cohort-level clustering with no per-fold equivalent, so the speaq feature columns were defined once on the whole cohort, whereas the binning and metabodeconplus features are re-derived inside every training fold; this favors speaq. For the same reason, a fitted speaq model cannot be applied to a new sample without re-clustering the cohort and re-training.

**Figure 6:**
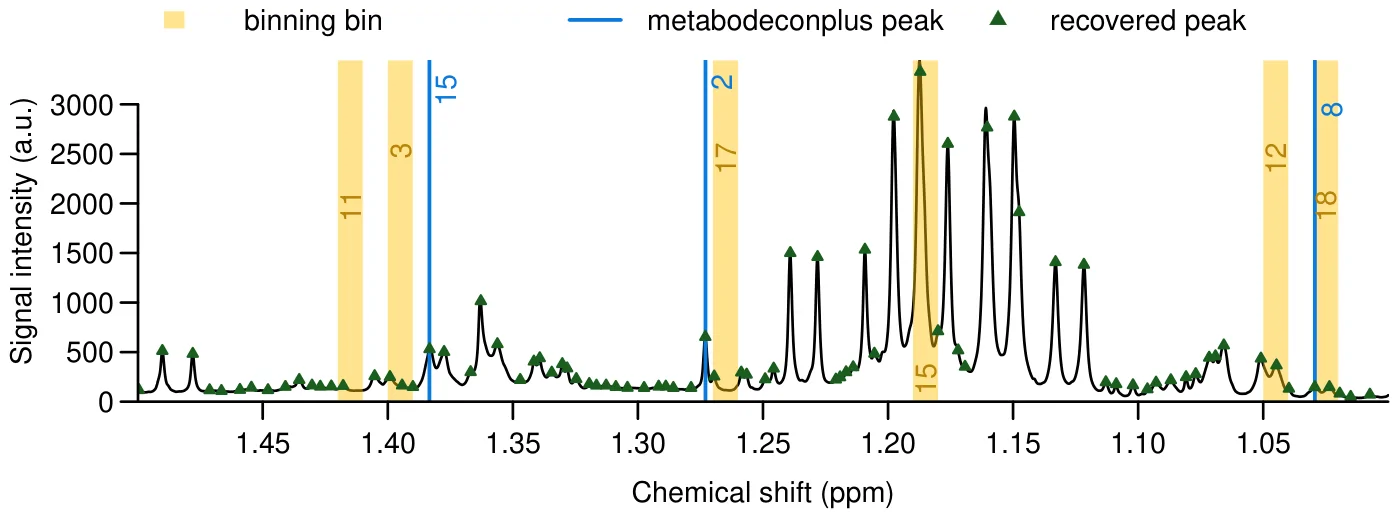
Display of informative features. Of the twenty most informative features of each model, the ones located in the 1.5–1.0 ppm section of the alignment reference spectrum are displayed. Orange bands mark bins of the binning baseline, blue lines mark peaks of the metabodeconplus model, and dark-green triangles mark every peak recovered from the reference spectrum. Labels give each feature’s rank in its model’s importance list. The remaining regions and the full ranked lists are in Appendix E.

To compare the features selected by the binning and metabodeconplus models, both were refitted on the full AKI dataset and their ranger permutation importances extracted. The top 20 features of each were overlaid on the alignment reference spectrum: Figure 6 shows the 1.5–1.0 ppm section, Appendix E the full range.

On its higher-resolution, per-signal feature matrix, metabodeconplus performed on par with the binning model. One main advantage of using a deconvolution approach is that each selected feature is localized at a single deconvoluted signal of the reference spectrum rather than spanning a fixed-width bin. This, in principle, allows for an unambiguous metabolite assignment of selected features. Note that this feature assignment step is not part of the metabodeconplus package. For assignment of deconvoluted signals, a comparison with database spectra of pure compounds measured under similar conditions is required. Once feature assignment has been done by external approaches, predictive signatures may be transferred to other methods from, for example, clinical chemistry. In addition, this allows for a biological interpretation of obtained signatures. In this context, it should be noted that predictive signatures only provide correlations between metabolites and groups of specimens; they do not provide causal relationships.

Whereas, as can be seen from Figure 6, for the binning model, a bin may contain contributions from multiple signals and metabolites, which substantially hinders assignment of predictive signatures to corresponding metabolites.

### 3.5. Runtime Performance and Parallel Scaling

**Figure 7:**
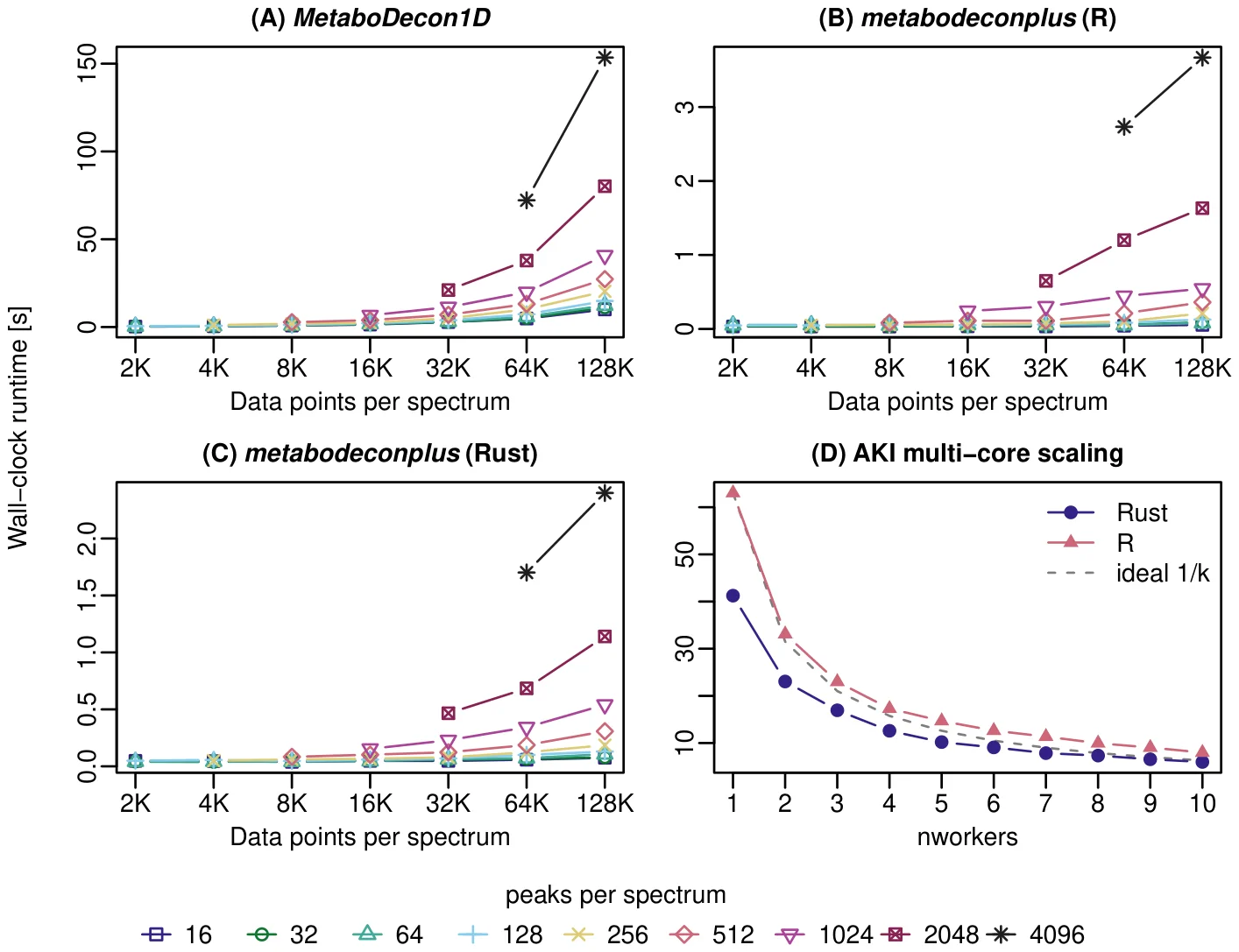
Runtime performance. (**A**–**C**) Single-core wall-clock for MetaboDecon1D, for the R implementation of metabodeconplus and for its Rust backend, over spectrum size and signal count; one line per signal count, shared key below the panels. (**D**) Wall-clock for deconvoluting all 106 AKI urinary spectra against the number of worker processes, per backend, with the dashed curve marking ideal 1/k scaling; parallelization is across spectra while each spectrum stays single-threaded. Timings are means over 5 repetitions per grid point; Appendix F gives the benchmark hardware.

The per-spectrum, single-core runtime of the original MetaboDecon1D implementation (Figure 7A); the R implementation of the new metabodeconplus package (Figure 7B); and the Rust backend of metabodeconplus as activated from within the R environment (Figure 7C) were compared. To this end, spectra with a different number of data points ($2^{11}$ to $2^{17}$) and signal counts ($2^{4}$ to $2^{12}$) were simulated. Typical 1D ^1^H spectra of biofluids such as urine and plasma consist of $2^{16}$ to $2^{17}$ data points with approximately 1000 signals. For the original MetaboDecon1D implementation, this resulted in an average runtime of around 40 s at $2^{17}$ data points (Figure 7A). In the R implementation of the new metabodeconplus package, the runtime is substantially reduced to well below one second per spectrum in this regime (Figure 7B). The Rust backend (Figure 7C) provides a further speed-up that becomes most pronounced for densely packed spectra: at $2^{17}$ data points and 4096 signals, the deconvolution takes around 3.7 s in pure R but only around 2.4 s with Rust, while at the more typical ≈1000 signals, the two backends are essentially indistinguishable on a single spectrum. Panels A–C of Figure 7 are single-core single-spectrum measurements; parallelization across spectra is shown separately in Figure 7D, showing the deconvolution times of all 106 AKI spectra with respect to the degree of parallelization (1 to 10 parallel workers) for both the R and Rust backend. Runtime scales close to the ideal 1/k reference throughout the measured range, with the Rust backend completing the full 106-spectrum batch in under 6 s on 10 workers and the R implementation in under 8 s. These short runtimes make it tractable to determine the optimal deconvolution parameters for each dataset individually by systematic grid search.

Because deconvolution is performed independently for each spectrum, with no shared state or inter-spectrum dependencies, total runtime scales linearly with the number of spectra, subject to the near-ideal 1/k speed-up of Figure 7D. A tenfold-replicated AKI cohort of 1060 spectra completed in 52 s with the Rust backend at nworkers=10, against the 60 s predicted by linear scaling from the 106-spectrum measurement (6.0 s), i.e., marginally faster than linear.

These figures cover deconvolution, the only stage that is independent per spectrum. Memory consumption is likewise linear in the size of the spectra. The per-peak bookkeeping cannot introduce a separate scaling term, since the number of detectable peaks is bounded by one third of the number of data points and is far lower in practice, while the storage per peak is a small constant. The total footprint therefore grows linearly with the number of spectra held in memory. Peak resident memory should be read as an upper bound rather than as the requirement of the algorithm, as R’s garbage collector does not deterministically return freed memory to the operating system. Measured on the replicated cohort for a single worker, where the peak is attributable to one process, the 1060 spectra occupy 2.1 GiB and the peak resident memory reaches 8.0 GiB, i.e., 3.8 times the size of the loaded spectra; repeated runs on identical input agreed to within 12%.

Because of R’s garbage collector, this is an upper bound rather than the requirement of the algorithm, and allows a workstation with 32 GiB of RAM to be used without swapping.

As a concrete example, the following costs were measured on the 106 AKI spectra with the pure-R backend, given as a single-core cost with the observed wall-clock time at nworkers=56 in parentheses. The 60-cell deconvolution grid that fit_mdm() evaluates once at entry costs 40 min (57 s), and alignment, the adaptive maxShift=−1 sweep plus the CluPA pass that follows it, costs 14 min (60 s). A complete fit_mdm() call with npmax=−1, maxShift=−1 and 5 maxCombine values costs 56 min (2.2 min). The single-core figures are CPU times of the parallel runs and therefore include parallelization overhead.

## 4. Discussion

With metabodeconplus, a single end-to-end workflow for the analysis of 1D NMR fingerprinting data of complex biofluids is provided, requiring no prior knowledge of the individual molecules present. The workflow integrates three steps that previously required separate tools: deconvolution of overlapping signals into individual Lorentzian lines, alignment of the deconvoluted signals, and sample classification, tied together by an automated parameter search. Results showed that, with the new implementation of the deconvolution algorithm, a substantial improvement in runtime could be obtained. With this, the deconvolution of large datasets containing several hundred spectra becomes feasible. This is especially true when the Rust implementation of the deconvolution part is used. This substantial improvement in runtime permits a systematic search of optimal deconvolution and alignment parameters. To analyze the performance of metabodeconplus in terms of signal detection and deconvolution, a carefully designed challenging simulated data set was used, where the ground truth is known. It was shown that metabodeconplus facilitates a reliable signal detection and deconvolution of overlapping signals in complex data. The residual gap to PRARPX=1 on the simulated data is dominated by small signals shadowed by larger neighbors (suppressing PRX); the additive-noise floor alone (raising AR above zero) caps PRARPX only at 0.999, as detailed in Section 3.1 and illustrated in Figure A2. Deconvolution quality depends on appropriate parameter selection. To this end, a grid search procedure was devised to search the parameter space by optimizing the Area Ratio. However, especially for complex experimental spectra, it is not guaranteed that this will always lead to an optimal selection. One critical parameter is the expected maximal number of signals npmax that ensures that there are not a large number of spurious Lorentzians that are fitted. For experimental data such as urine and plasma spectra, the maximum number of expected signals can be set from prior knowledge, or left to the elbow heuristic of Appendix G by passing npmax=−1. To make full use of the deconvoluted data in subsequent statistical analysis proper signal alignment is required to correct for small variations in signal positions across spectra. For alignment, the well-established CluPA algorithm is employed. It was extended by the snap_to_ref function to ensure that corresponding peaks are put to the exact same position. As was shown, this in general provides good alignment. However, alignment quality depends on the two parameters maxShift and maxCombine. These, together with npmax, may be manually set by the user after careful spectra inspection. In addition, an automated supervised grid search via fit_mdm() was devised that in principle allows all three parameters to be tuned jointly. For this, two well-separable classes with known class labels are required. In practice, it is recommended, as mentioned above, to fix npmax from domain knowledge or visual inspection or automatically based on the so-called elbow heuristic, leaving maxShift to the adaptive CluPA-internal sweep, and to tune only maxCombine on the labeled data; this avoids the multiplicative runtime of the full three-dimensional grid without sacrificing classification performance. The higher spectral resolution and interpretability of the signal-based feature matrix were achieved without loss of predictive performance. The classification performance of metabodeconplus matched the equidistant-binning baseline. This, in principle, allows each discriminatory feature to be assigned to an individual metabolite. This is often not possible with a coarse bin, which potentially contains contributions from several signals. The cross-sample peak groups of speaq 2.0 [11] classified as well as either of them (Section 3.4), but a peak group, like a bin, is a position on the chemical-shift axis without a fitted line shape, and it is defined on the cohort analyzed, so a speaq model does not transfer to a new sample without re-clustering.

Limitations: Direct file-format support is restricted to Bruker and JCAMP-DX; other vendor formats are not parsed natively. Users can, however, provide spectra as a chemical-shift vector and a corresponding signal-intensity vector, so any format that can be loaded into the R session is usable. Native readers for further vendor formats remain as future work. No routines are provided to group deconvoluted signals into multiplet structures, and no automatic signal-to-metabolite assignment (e.g., matching against reference spectra) is included. Finally, PRARPX has a hard upper bound below 1 on noisy spectra: additive measurement noise keeps AR>0, and signals completely overlapped by larger neighbors produce no detectable curvature minimum and are therefore not recovered (Figure A2). The set of experimental data sets used for testing the metabodeconplus package is limited. However, the urinary AKI dataset used represents a challenging test case. In comparison to serum, plasma, and tissue extracts, urine spectra generally contain more signals, leading to increased signal overlap, which makes them a real challenge for deconvolution. Furthermore, signal shifts across spectra are usually larger in urine than in serum, plasma, or tissue extracts, making urine a challenging matrix for signal alignment. Therefore, it is safe to expect that metabodeconplus will also perform well for other sample matrices. However, a broader validation employing additional urine and plasma cohorts would clearly strengthen this claim. All spectra used here were measured at a ^1^H resonance frequency of 600 MHz. For spectra measured at substantially lower resonance frequencies, increased signal overlap is expected, which in some cases might lead to complete shadowing and therefore incomplete deconvolution of some smaller signals.

## 5. Conclusions

In summary, metabodeconplus makes 1D NMR fingerprinting a single automated workflow (from deconvolution through alignment to classification) with automated parameter optimization and a fast, parallelized backend that makes this end-to-end pipeline practical at the scale of modern metabolomics cohorts. For example, metabodeconplus could be applied to questions like the integrated use of microbiome and urinary metabolome data to predict secondary infection in critically ill patients, as described in [34].

## Figures and Tables

**Table 1:** Summary of tasks performed by various published approaches. *^a^* Indicated are only approaches that perform a dedicated signal deconvolution of overlapping signals. *^b^* Tools that allow an absolute quantification of metabolites. *^c^* Approaches performing a dedicated signal alignment, allowing for non-linear signal corrections, across a set of measured spectra. *^d^* Additional tools to perform a statistical analysis of obtained data. *^e^* Commercial approaches.

Name	Deconvolution *^a^*	Quantification *^b^*	Alignment *^c^*	Statistics *^d^*
ACD/NMR *^e^*	✓	✓	–	✓
AMIX *^e^*	✓	✓	–	✓
ASICS	✓	✓	–	✓
BATMAN	✓	✓	–	–
BAYESIL	✓	✓	–	–
Chenomx *^e^*	✓	✓	–	–
COW	–	–	✓	–
decon1d	✓	–	–	–
DEEP Picker1D	✓	–	–	–
icoshift	–	–	✓	–
metabodeconplus	✓	✓	✓	✓
MetaboDecon1D	✓	–	–	–
mldecon	✓	–	–	✓
Mnova NMR *^e^*	✓	✓	–	✓
NMRProcFlow	–	–	✓	✓
rDolphin	✓	✓	–	–
SigMa	✓	✓	✓	–
speaq 2.0	✓	–	✓	✓

**Table 2:** Summary of sample types, number of samples, and measurement techniques used. The Blood dataset seeds the reference signal parameters of the simulated Sim3 dataset (Section 3.1); the Urine dataset is used to select the default npmax for the AKI benchmark (Section 3.4).

Name	Sample Type	Number of Samples	Exp. Technique
Sim3	Simulated	100	1D Sim
Blood	Human Blood Plasma	16	1D CPMG
Urine	Human Urine	2	1D NOESY
AKI	Human Urine	106	1D NOESY

**Table 3:** PRARPX summary on the Sim3 dataset across all spectra: mean, standard deviation, minimum, and maximum for each configuration. Bold marks the best achievable (mean, SD) pair (highest mean, ties broken by smallest SD); the metabodeconplus optimal row is excluded from this comparison.

Configuration	Mean	SD	Min	Max
MetaboDecon1D (default)	0.712	0.041	0.610	0.814
metabodeconplus (default)	0.791	0.037	0.691	0.872
metabodeconplus (npmax = 10)	0.734	0.045	0.593	0.833
metabodeconplus (npmax = 15)	0.734	0.045	0.593	0.833
metabodeconplus (npmax = 20)	0.751	0.030	0.661	0.833
metabodeconplus (npmax = 25)	**0.801**	**0.041**	0.693	0.876
metabodeconplus (npmax = 30)	0.796	0.044	0.654	0.876
metabodeconplus (npmax = 35)	0.796	0.044	0.654	0.876
metabodeconplus (npmax = 40)	0.796	0.044	0.654	0.876
metabodeconplus (optimal)	0.812	0.032	0.745	0.876

**Figure A1:**
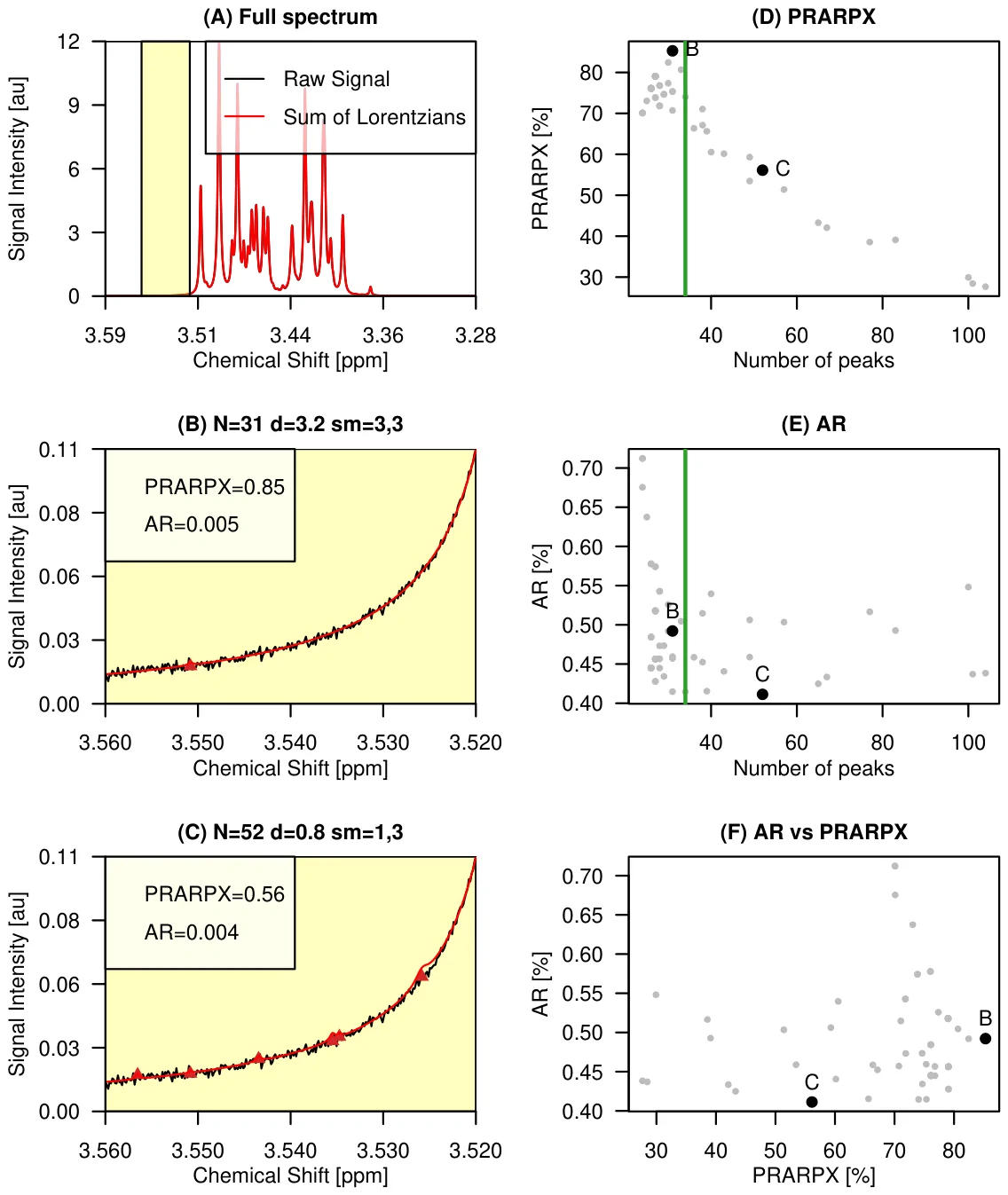
Performance metrics for evaluating deconvolution quality on a simulated spectrum with 34 true signals. (**A**) The full observed spectrum; the yellow rectangle marks the chemical-shift window, 3.520–3.560 ppm, shown in panels (**B**,**C**). In panels (**B**,**C**), red triangles mark spurious fitted peaks (false positives). (**B**) The grid configuration with the highest PRARPX. It fits 31 peaks (30 match a true signal, 1 is spurious), yielding PRARPX=0.85 and AR=0.005. (**C**) The grid configuration with the lowest AR. It fits 52 peaks (31 match a true signal, 21 are spurious): the residual area is smaller than in (**B**) (AR=0.004), but the spurious peaks drag PRARPX down to 0.56. In total, 41 of 66 grid configurations achieve a strictly lower AR than configuration (**B**), illustrating that AR alone does not identify the best deconvolution. (**D**) PRARPX over the full grid: PRARPX peaks near the true number of peaks (green vertical line at 34) and decays for both under- and overfitted configurations. (**E**) AR over the same grid: AR keeps decreasing as the number of peaks grows past the truth, so AR alone is not a reliable indicator of deconvolution quality. (**F**) Scatter of AR versus PRARPX: high PRARPX implies low AR, but low AR does not imply high PRARPX. Overfitted configurations form a cluster at low PRARPX whose AR overlaps that of the high-PRARPX configurations.

**Figure A3:**
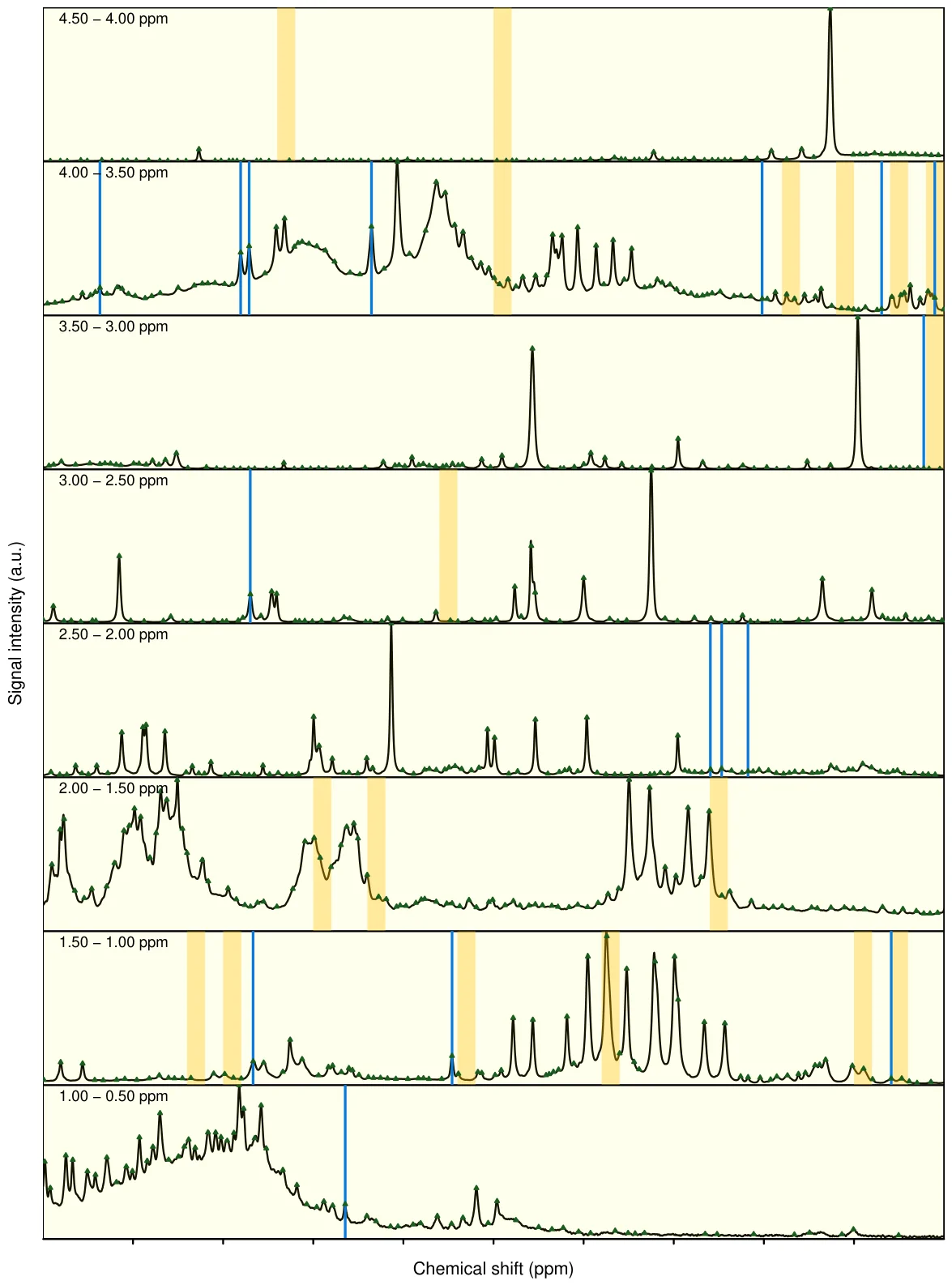
The twenty most informative features of each model over the aliphatic 4.5–0.5 ppm region in eight rows of 0.5 ppm. Colors are those of Figure 6.

**Figure A4:**
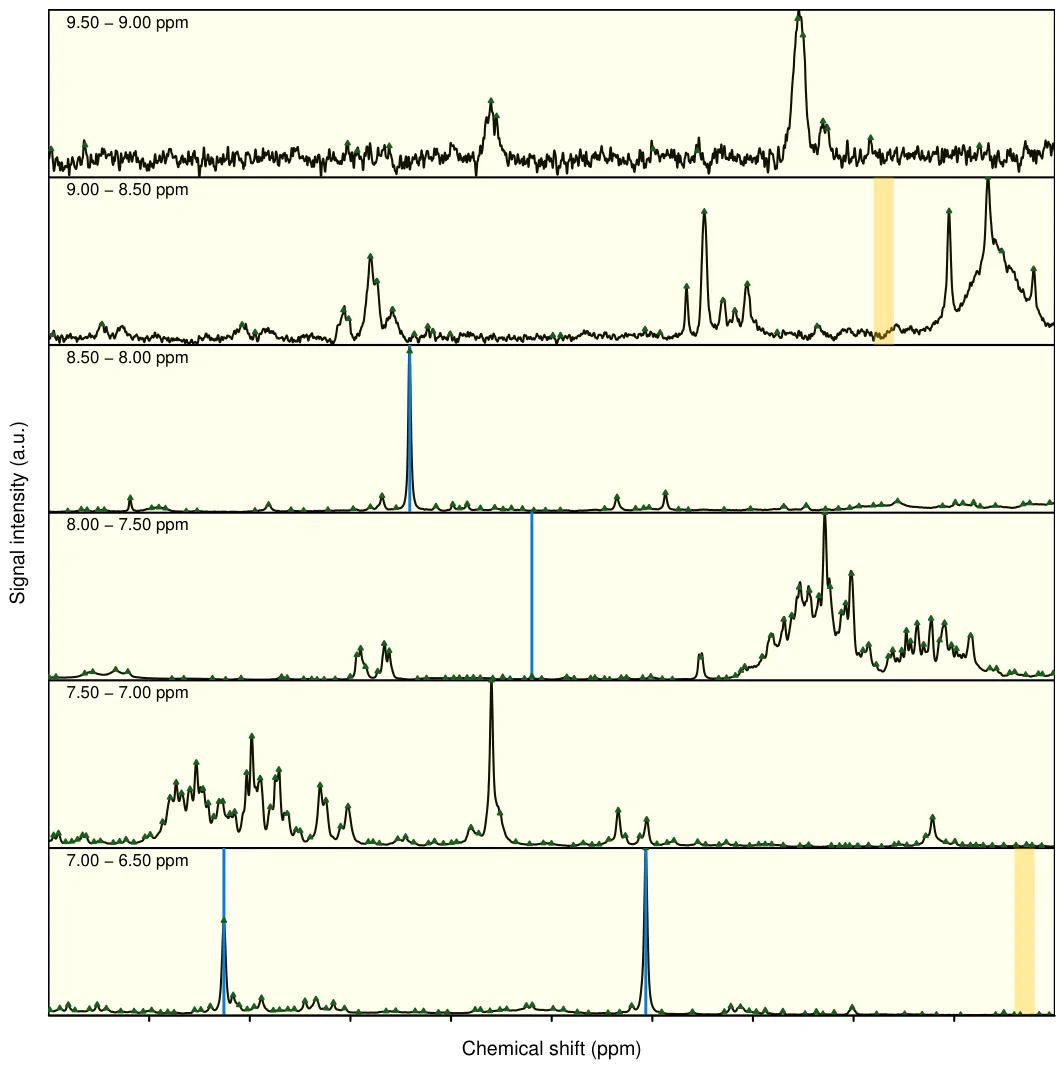
Aromatic-region counterpart to Figure A3, showing the 9.5–6.5 ppm window. Of the twenty highest-ranked features per model, two bins and four metabodeconplus signals are located in this window; the remaining features are located in the aliphatic region of Figure A3. Layout and colors match Figure A3: orange bands mark the bins selected by the binning model, blue vertical lines mark the peaks selected by the metabodeconplus model, and dark-green triangles mark every peak that metabodeconplus recovered from the reference spectrum.

**Figure A6:**
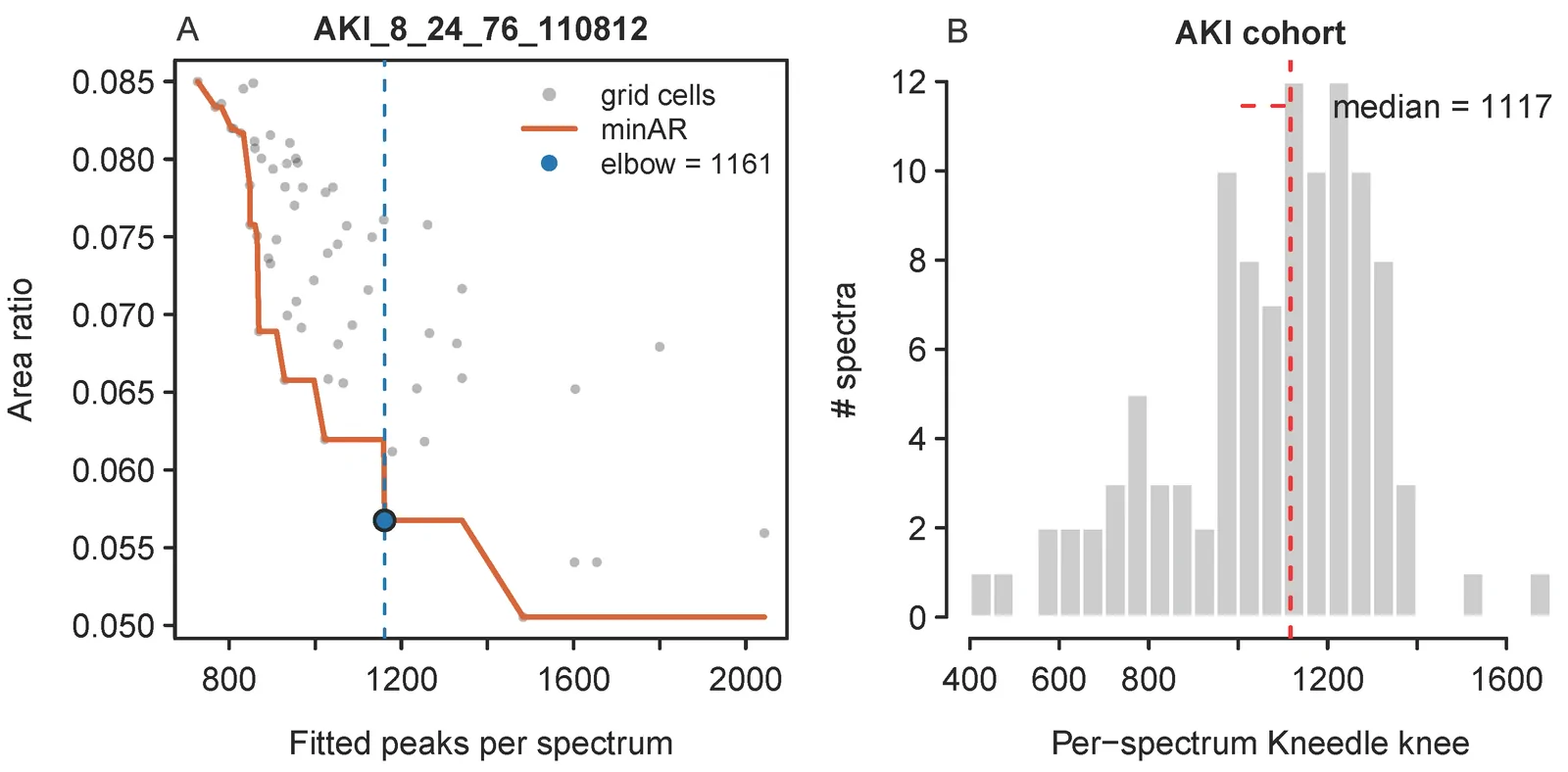
(**A**) Area ratio against number of fitted signals for one AKI spectrum. Gray markers are the 60 cells of the parameter grid; minAR is the minimal achievable AR at each npmax, and the filled point is its elbow. (**B**) Elbows of all 106 AKI spectra; dashed line, their median.

**Figure A7:**
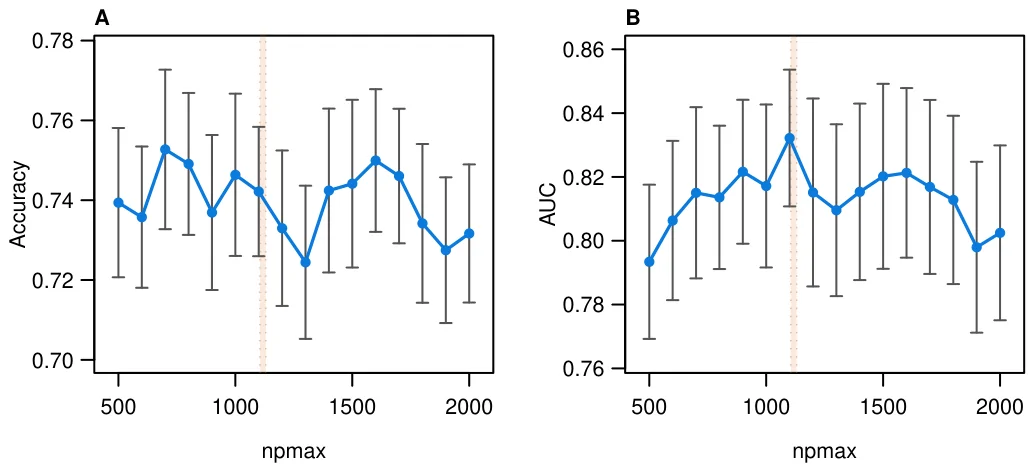
Cross-validated accuracy (**A**) and AUC (**B**) of the AKI benchmark against npmax. Points are means over 30 folds, and bars are one standard error. The shaded band spans the budgets npmax=−1 resolved to across those folds.

## Data Availability

Publicly available datasets were analyzed in this study. The AKI, Blood and Urine datasets are available from the metabodeconplus GitHub repository at https://github.com/spang-lab/metabodeconplus (accessed on 11 August 2026); the simulated Sim3 dataset is regenerated deterministically by the analysis code accompanying this manuscript. The urinary AKI dataset has also been uploaded to the publicly available MetaboLights database [35], identifier: MTBLS24. The GCKD plasma spectra are also available from the MetaboLights database, identifier: MTBLS798. All results reported here were produced with metabodeconplus versions 0.20.0 to 0.22.2 and the random seeds 1, 2 and 3 for every repeated cross-validation, using the scripts shipped with the accompanying mdpp package. The code that reproduces all figures and tables of this manuscript is available as mdpp.zip, attached to the 0.22.0 GitHub release of metabodeconplus and downloadable from https://github.com/spang-lab/metabodeconplus/releases/download/v0.22.0/mdpp.zip (accessed on 11 August 2026).

## Abbreviations

The following abbreviations are used in this manuscript:

1D	one-dimensional
AKI	acute kidney injury
AR	Area Ratio
AUC	area under the receiver operating characteristic curve
CluPA	cluster-based peak alignment
CPB	cardiopulmonary bypass
CPMG	Carr–Purcell–Meiboom–Gill
CRAN	Comprehensive R Archive Network
CSF	cerebrospinal fluid
CV	cross-validation
GCKD	German Chronic Kidney Disease
NMR	nuclear magnetic resonance
NOESY	nuclear Overhauser effect spectroscopy
OOB	out-of-bag
pp	percentage points
PR	Peak Ratio
PRARPX	product of PRX and 1−AR
PRX	Extended Peak Ratio
SD	standard deviation
SVM	support-vector machine

## Appendix A. Existing Solutions for NMR Spectra Analysis

1. ACD/NMR Workbook Suite by ACD/Labs is a commercial software suite for NMR data processing. It includes spectral deconvolution, metabolite quantification, and statistical analysis capabilities, but the underlying algorithms are proprietary and not publicly disclosed.
2. AMIX by Bruker is a commercial software suite for NMR-based metabolomics. It supports spectral processing, bucketing, and statistical analysis, but its deconvolution and alignment algorithms are proprietary.
3. Chenomx NMR Suite by Chenomx Inc. is a commercial software suite combining spectral deconvolution and library-based metabolite identification and quantification. Implementation details are not publicly available.
4. Mnova NMR by Mestrelab Research is a commercial software suite for NMR data processing, offering deconvolution, quantification, and statistical analysis. Its algorithms are proprietary.
5. MetaboLab [36] is a MATLAB-based software for NMR data processing offering algorithms for baseline correction and alignment via a graphical user interface. The software appears to no longer be actively maintained.
6. BATMAN [9] uses Bayesian modeling and Markov-Chain Monte Carlo (MCMC) together with spectral libraries for automated metabolite quantification in 1D NMR spectra. It incorporates prior signal information and handles overlaps and baseline distortions but requires careful tuning and substantial computational resources.
7. BAYESIL [18] is a fully automated web system for rapid NMR spectral profiling. Given a 1D ^1^H NMR spectrum of a complex biofluid, it autonomously identifies and quantifies metabolites with high accuracy using Bayesian spectral fitting against a reference library.
8. icoshift [16] is a MATLAB application for alignment of 1D NMR spectra. It optimizes the cross-correlation between user-defined intervals of a target and a reference spectrum to correct chemical-shift variations. SigMa [13] uses a modified version of the icoshift algorithm for its alignment step.
9. COW (Correlation Optimized Warping) [17] is a MATLAB application for chromatographic and spectroscopic data alignment. It divides the spectrum into segments and uses dynamic programming to maximize the segment-wise cross-correlation between a sample and a reference, allowing non-linear warping of the chemical-shift axis.
10. decon1d [15] is a Python script for deconvoluting 1D NMR spectra, originally developed for ^19^F spectra of labeled proteins. It iteratively places peaks using the Levenberg–Marquardt algorithm and selects the most parsimonious model via the Bayesian Information Criterion (BIC).
11. NMRProcFlow [20] is a graphical and interactive web application for preprocessing 1D NMR spectra, covering baseline correction, alignment, and bucketing. It does not include a dedicated signal deconvolution algorithm but applies a Least-Squares approach for alignment of user-defined intervals.
12. AQuA [37] is a software tool for automated quantification of metabolites in 1D NMR spectra, addressing signal overlap and baseline distortions.
13. rDolphin [10] is an R package for analysis of 1D NMR spectra that combines spectral library fitting for metabolite quantification with interactive optimization capabilities.
14. ASICS [12] is an R package providing a complete workflow for 1D ^1^H NMR spectra. For quantification, it first aligns selected library spectra with the sample spectrum, then fits the aligned library spectra using a sparse model.
15. NMRbox [38] is a web platform offering virtual machines with a broad collection of NMR software tools. It does not provide its own standalone deconvolution or alignment algorithm.
16. speaq 2.0 [11] is an R package for high-throughput processing of 1D NMR spectra. Signals are first represented as wavelets and then aligned across spectra using the hierarchical CluPA algorithm [22], yielding a two-dimensional feature matrix suitable for downstream statistical analysis with tools such as MetaboAnalyst [39].
17. SigMa [13] is a fully automated approach for quantification of 1D ^1^H NMR metabolomics data, particularly from human urine. It combines peak picking, a modified icoshift alignment, and signal deconvolution and explicitly discriminates between signals matching known reference metabolites and unassigned spectral regions.
18. MetaboDecon1D [14] is an R package for automatic deconvolution of 1D NMR spectra into Lorentzian curves using a curvature-based peak-detection algorithm. It is the direct predecessor of metabodeconplus.
19. DEEP Picker1D [19] is a convolutional neural network trained on synthetic 1D NMR spectra for signal detection and parameter estimation. Predicted signal parameters are refined by a Voigt fitter via nonlinear least squares, yielding a full quantitative representation of the spectrum.
20. mldecon [21] is a deep-learning-based deconvolution command available in Bruker TopSpin 4.2. Trained on synthetic spectra, it accurately estimates signal parameters and performs well on crowded, high-dynamic-range, and shoulder-peak regions.

## Appendix B. Major Differences Between the Current Version and MetaboDecon1D

**Table A1:** Concise comparison of major functional differences between MetaboDecon1D and metabodeconplus.

Aspect	MetaboDecon1D	Metabodeconplus
Scope	Deconvolution	Deconvolution, Alignment, Modeling
Implemented in	R	R and Rust
Lorentzian fitting	Uses original formulation	Uses algebraically simplified equations
Smoothing	Smoothed intensities propagate into fitting	Smoothing is used for peak detection only; Lorentzian fitting uses the raw intensities
Artifact handling	Negative intensities rectified; water region set to zero	Negative intensities retained; user-defined ignore regions replace hard zeroing of artifact regions
Parameter optimization	Manual parameter selection	Manual selection or grid search via npmax
Alignment	Not available	CluPA followed by snap_to_ref
Predictive modeling	Not available	End-to-end classifier pipeline via fit_mdm() with a Random Forest learner; nested-CV performance estimation via benchmark()
Performance and reuse	Legacy implementation	Faster peak detection and smoothing, parallel execution, and optimized Lorentzian superposition

## Appendix C. Mathematical Details of Lorentzian Function Fitting

The Lorentzian function, also known as the probability density function of the Cauchy distribution, is defined as

(A1) $$f\left(\omega ;\omega_{0},\lambda \right)=\frac{1}{\pi }\frac{\lambda }{{\left(\omega -\omega_{0}\right)}^{2}+\lambda^{2}}$$

where $\omega_{0}$ is the center and λ is the half-width at half-maximum. Since the integral is normalized to 1, an additional amplitude factor *A* replaces the normalization term $\frac{1}{\pi }$:

(A2) $$f\left(\omega ;\omega_{0},\lambda ,A\right)=A\cdot \frac{\lambda }{{\left(\omega -\omega_{0}\right)}^{2}+\lambda^{2}}$$

For each detected signal, a stencil of three observed points $\left(\omega_{i},y_{i}\right)$, i=1,2,3, gives the system

(A3) $$y_{i}=A\cdot \frac{\lambda }{{\left(\omega_{i}-\omega_{0}\right)}^{2}+\lambda^{2}},\;i=1,2,3$$

Eliminating *A* and λ yields a closed-form expression for $\omega_{0}$:

(A4) $$\omega_{0}=\frac{1}{2}\frac{\omega_{1}^{2}y_{1}\left(y_{2}-y_{3}\right)+\omega_{2}^{2}y_{2}\left(y_{3}-y_{1}\right)+\omega_{3}^{2}y_{3}\left(y_{1}-y_{2}\right)}{y_{1}y_{2}\left(\omega_{1}-\omega_{2}\right)+y_{2}y_{3}\left(\omega_{2}-\omega_{3}\right)+y_{3}y_{1}\left(\omega_{3}-\omega_{1}\right)}$$

With $\omega_{0}$ known, the product A·λ can be isolated:

(A5) $$A\cdot \lambda =y_{i}\cdot \left({\left(\omega_{i}-\omega_{0}\right)}^{2}+\lambda^{2}\right),\;i=1,2,3$$

Comparing any two instances of Equation (A5) gives an expression for $\lambda^{2}$:

(A6) $$\lambda_{jk}^{2}=\frac{y_{k}{\left(\omega_{k}-\omega_{0}\right)}^{2}-y_{j}{\left(\omega_{j}-\omega_{0}\right)}^{2}}{y_{j}-y_{k}},\;j,k\in \left\{1,2,3\right\},\;j\neq k$$

Using the outer pair (j=1,k=3) directly causes numerical instability because quasi-symmetric signals have $y_{1}\approx y_{3}$. Instead, $\lambda_{12}^{2}$ and $\lambda_{23}^{2}$ are computed and averaged:

(A7) $$\lambda^{2}=\frac{1}{2}\left(\lambda_{12}^{2}+\lambda_{23}^{2}\right)$$

The product A·λ is then obtained from the central point i=2:

(A8) $$A\cdot \lambda =y_{2}\cdot \left({\left(\omega_{2}-\omega_{0}\right)}^{2}+\lambda^{2}\right)$$

Note that A·λ together with $\omega_{0}$ and $\lambda^{2}$ is sufficient to evaluate the Lorentzian at any ω without resolving *A* and λ separately.

After computing initial estimates for all Lorentzians, the superposition is compared to the raw unsmoothed intensities. Following Koh et al. [27], the intensity stencils of each Lorentzian are rescaled by the ratio of observed to reconstructed intensity (rule of proportion), and the fitting step is repeated. This iterative refinement continues for a user-defined number of iterations.

In the original MetaboDecon1D implementation [14], smoothed intensities were used for both peak detection and fitting. Smoothing disproportionately reduces the apparent height and width of narrow signals, biasing the parameter estimates. metabodeconplus uses smoothed intensities only for signal detection and fits Lorentzian parameters to the raw intensities, reducing this systematic bias. Additionally, estimated *A* and λ values below a multiple of machine precision are discarded at the end of refinement to suppress numerical artifacts.

## Appendix D. The PRARPX Metric

Common methods for assessing deconvolution quality include evaluation of the Area Ratio (AR); evaluation of the Peak Ratio (PR), i.e., comparing the number of detected signals to the true number of signals; and visual inspection. However, all of these methods have limitations. Residual-only criteria such as the AR tend to improve with the number of detected signals, regardless of the true number of signals. Finding the correct number of signals does not necessarily mean that the correct signal parameters have been found. Additionally, in real-world applications, the true number of signals is typically unknown. Visual inspection is subjective and not suitable for large datasets. PRARPX was developed for evaluating deconvolution quality when true signals are known, as is the case for simulated data or real samples with spiked-in controls.

Let $T=\left\{t_{1},\ldots ,t_{n_{T}}\right\}\neq \emptyset$ denote the true components that generate the spectrum, and $D=\left\{d_{1},\ldots ,d_{n_{D}}\right\}\neq \emptyset$ the fitted components recovered by deconvolution. The Jaccard index is a common metric for comparing two sets:

(A9) $$J\left(T,D\right)=\frac{\left|T\cap D\right|}{\left|T\cup D\right|}=\frac{\left|T\cap D\right|}{\left|T|+|D|-|T\cap D\right|}.$$

However, given components are points in continuous parameter space, T∩D=∅ almost surely and J(T,D)=0 irrespective of fit quality. The Jaccard index is therefore uninformative in this setting. Now, let

(A10) $$\varphi :D\to T,\;\varphi \left(d\right)=\arg\min_{t\in T}\left|\omega_{0,d}-\omega_{0,t}\right|.$$

By the same argument, a fitted component is exactly equidistant from two true components only on a null set, so φ is almost surely independent of how ties are broken. The image of this map, $\varphi \left[D\right]=\left\{\varphi (d)|d\in D\right\}$, corresponds to the set of true components whose preimage is not the empty set under the map. Further, φ is a bijection if and only if |T|=|D| and each true component’s preimage contains at least one fitted component under the map, in which case φ[D]=T, and thereby |φ[D]|=|T|=|D|. We can then define a modified Jaccard index, the Extended Peak Ratio (PRX):

(A11) $$\mathrm{PRX}\left(T,D\right)=\frac{\left|\varphi [D]\right|}{\left|T|+|D|-|\varphi [D]\right|}.$$

Write $k_{r}=\left|\varphi \left[D\right]\right|$ for the number of true components recovered, so that

(A12) $$\mathrm{PRX}\left(T,D\right)=f\left(k_{r}\right),\;f\left(k_{r}\right)=\frac{k_{r}}{\left|T\right|+\left|D\right|-k_{r}}.$$

Since φ[D]⊆T and φ[D] is the image of a map defined on *D*, we have $k_{r}\leq \left|T\right|$ and $k_{r}\leq \left|D\right|$, and since D≠∅, it also holds that $k_{r}\geq 1$. Abbreviating m=min(|T|,|D|) and M=max(|T|,|D|), this gives $1\leq k_{r}\leq m$. On this range, the denominator of *f* is bounded below by

(A13) $$\left|T\right|+\left|D\right|-k_{r}\;\geq \;m+M-m\;=\;M\;>\;0,$$

so *f* is well defined, and it is strictly increasing in $k_{r}$, as for $1\leq k_{r}<k_{r}^{\prime }\leq m$, both $k_{r}<k_{r}^{\prime }$ and $\left|T\right|+\left|D\right|-k_{r}>|T|+\left|D\right|-k_{r}^{\prime }$, which implies that $f\left(k_{r}\right)<f\left(k_{r}^{\prime }\right)$. The extreme values of *f* on {1,…,m} are therefore attained at the boundaries $k_{r}=1$ and $k_{r}=m$, giving

(A14) $$0<\frac{1}{|T|+|D|-1}=f\left(1\right)\leq \mathrm{PRX}\left(T,D\right)\leq f\left(m\right)=\frac{m}{m+M-m}=\frac{m}{M}\leq 1,$$

and therefore PRX∈(0,1]. The lower bound is strictly positive for any finite |T| and |D|, but f(1)→0 as |T|+|D|→∞, so PRX can be made arbitrarily small. Since PRX≤m/M, the score is capped by the imbalance between |T| and |D| alone, irrespective of how well the two sets are matched. If the ground truth contains many components that are not recoverable, whether through strong overlap or intensity vanishing against the noise level, then |D|≪|T| and PRX≤|D|/|T|; conversely, if the spectrum is noisy enough that many artifacts are introduced, then |D|≫|T| and PRX≤|T|/|D|. In both cases, the attainable score degrades in proportion to the imbalance, even for an otherwise perfect matching of the components that are present in both sets.

As a second component, the PRARPX metric incorporates a measure of agreement between the area of the observed spectrum and the reconstructed spectrum. To this end, we define the Area Ratio (AR):

(A15) $$\mathrm{AR}=\frac{\sum_{i=1}^{N}\left|Y\left(\omega_{i}\right)-\hat{Y}\left(\omega_{i}\right)\right|}{\sum_{i=1}^{N}\left|Y(\omega_{i})\right|}=\frac{R}{S},$$

where S>0. Note that *R* and *S* are $\ell^{1}$ norms rather than areas. On a uniformly spaced grid with spacing Δω, however, both sums carry the same quadrature weight Δω, which cancels in the quotient, so AR is exactly the ratio of the corresponding areas. It is easy to see that, generally, AR can grow arbitrarily large and $\mathrm{AR}\in R_{\geq 0}$, where AR=0 corresponds to perfect agreement between the observed and reconstructed spectra, and AR>1 corresponds to a residual greater than the observed signal. In practice, the latter is exceedingly unlikely to occur outside of implementation errors in a fitting algorithm.

As such, we will first consider the case R≤S, where AR∈[0,1] holds trivially. Note that, unlike PRX, a value closer to 0 corresponds to better agreement. That is to say, the orientation is inverted relative to PRX. Further, for the R>S case, AR∉[0,1]. To correct for these issues, we subtract AR from 1 and restrict the result to a minimum of 0, yielding max(0,1−AR)∈[0,1]. As reasoned above, though, the lower bound is unlikely to ever be reached in practice, and so is the upper bound due to noise. In order to combine both of these metrics, we use their product, which we call the PRARPX metric:

(A16) PRARPX=PRX·max(0,1−AR).

Since both factors are in [0,1], it also follows that PRARPX∈[0,1]. It further holds that the optimal value PRARPX=1 is achieved if and only if φ is a bijection and R=0, i.e., the fitted components match the true components one-to-one, and the reconstructed spectrum perfectly matches the observed spectrum. Conversely, PRARPX=0 holds if and only if R≥S, since PRX>0 always. A vanishing score is therefore attributable to the residual alone. The value 0 can nonetheless be approached arbitrarily closely through PRX, by taking |T|+|D|→∞ while $k_{r}\in o(|T|+\left|D|\right)$, i.e., the number of distinct true components recovered grows asymptotically more slowly than the total number of components.

A visualization of the above-mentioned metrics (AR, PR, PRX, and PRARPX) for one example simulated spectrum is shown in Figure A1. The spectrum has been deconvoluted using different deconvolution parameters, resulting in different deconvolution results. As evident from Figure A1B,D, an increasing number of peaks used for deconvolution usually leads to decreasing AR values even when the number of true signals has been considerably exceeded. Therefore, using the AR alone to compare deconvolution quality in controlled benchmark settings may not be optimal in all cases. In contrast, PRARPX reaches high values only when both the spectral approximation is good and the correct number of signals has been recovered. Thus, PRARPX is a more informative metric for comparing deconvolution quality in scenarios where the ground truth is known, such as simulated data or controlled measurements of known compounds. However, it is not suitable as an investigative metric for ordinary experimental data, where the true signal parameters are unknown.

## Appendix E. End-to-End AKI Benchmark: Supplementary Outputs

This appendix lists the discriminative features of the three models of Section 3.4 (binning, speaq 2.0 and metabodeconplus), two of which are shown in Figure 6. All three were trained on the full AKI dataset, and their features were ranked by permutation importance. This is defined as the loss in classification accuracy that results from randomly shuffling a feature’s value across samples, averaged across the trees of the forest. Table A2 reports the twenty highest-ranked features of the binning approach, speaq 2.0 and metabodeconplus, together with their chemical-shift positions and importance scores. Figure A4 is the aromatic-region counterpart to Figure 6: of the twenty highest-ranked features per model, two bins and four metabodeconplus peaks fall in the aromatic 9.5–6.5 ppm window, while the remainder occupy the aliphatic region of Figure A3.

**Table A2:** The twenty highest-ranked features of the three models, each with ranger on the full AKI dataset: 700 equidistant bins, speaq 2.0 peak groups (wavelet peak detection, peak grouping and feature-matrix construction) and the metabodeconplus pipeline (deconvolute → CluPA →snap_to_ref) at the combination selected by fit_mdm() (npmax=−1, maxShift auto-tuned, maxCombine∈{2,4,8,16,32}). Center refers to the center of the 0.01 ppm-wide bin, the chemical shift of the cross-sample peak group and the chemical shift of the peak on the alignment reference, respectively; Imp. is the permutation-importance score.

Binning			Speaq 2.0			Metabodeconplus		
**Rank**	**Center**	**Imp.**	**Rank**	**Center**	**Imp.**	**Rank**	**Center**	**Imp.**
1	3.505	0.0038	1	6.5162	0.0057	1	2.1088	0.0040
2	3.525	0.0025	2	1.2722	0.0035	2	1.2730	0.0039
3	1.395	0.0023	3	1.0441	0.0034	3	6.9129	0.0037
4	1.815	0.0018	4	2.4070	0.0024	4	2.8849	0.0024
5	1.625	0.0016	5	1.3987	0.0021	5	3.5052	0.0024
6	4.245	0.0015	6	1.0292	0.0021	6	3.9684	0.0022
7	3.555	0.0015	7	1.3769	0.0018	7	8.3205	0.0021
8	3.745	0.0014	8	3.1968	0.0017	8	1.0293	0.0020
9	4.365	0.0014	9	6.9158	0.0016	9	0.8323	0.0020
10	2.775	0.0013	10	1.3829	0.0015	10	3.8856	0.0018
11	1.415	0.0013	11	2.1429	0.0014	11	3.8903	0.0017
12	1.045	0.0013	12	1.8489	0.0014	12	3.6010	0.0017
13	3.585	0.0012	13	3.0112	0.0013	13	6.7032	0.0015
14	6.515	0.0011	14	4.4484	0.0013	14	2.1297	0.0014
15	1.185	0.0011	15	1.6245	0.0013	15	1.3834	0.0014
16	8.585	0.0011	16	7.3088	0.0013	16	3.5347	0.0013
17	1.265	0.0011	17	2.8842	0.0013	17	7.7598	0.0013
18	1.025	0.0011	18	3.4738	0.0013	18	2.1234	0.0012
19	3.005	0.0011	19	2.8718	0.0013	19	3.0113	0.0011
20	1.845	0.0010	20	1.6488	0.0012	20	3.8177	0.0011

**Table A3:** Fold-wise paired comparison of the three feature representations over the 30 shared folds. Δ is the mean difference, with a paired *t*-test and Holm correction across the family.

Comparison	Metric	Δ	95% CI	*t*-Test	Wilcoxon
metabodeconplus vs. binning	Accuracy (pp)	−1.7	[−6.2,+2.9]	1.00	0.96
metabodeconplus vs. speaq 2.0	Accuracy (pp)	−2.9	[−6.5,+0.8]	0.69	0.80
speaq 2.0 vs. binning	Accuracy (pp)	+1.2	[−1.5,+3.9]	1.00	0.96
metabodeconplus vs. binning	AUC	+0.017	[−0.020,+0.053]	1.00	0.96
metabodeconplus vs. speaq 2.0	AUC	+0.013	[−0.019,+0.044]	1.00	0.96
speaq 2.0 vs. binning	AUC	+0.004	[−0.018,+0.026]	1.00	0.96

## Appendix F. CPU Specifications for Benchmarking

All benchmarks were executed on a dual-socket server with two Intel Xeon Gold 6348 processors, providing 28 physical cores per socket (56 total) and, with hyperthreading enabled, 112 logical threads. The system provided 1 TiB of RAM. Topology and cache details are summarized in Table A4.

**Table A4:** Benchmark hardware specification.

Property	Value
CPU	2× Intel Xeon Gold 6348
Microarchitecture	Ice Lake-SP
Cores per socket/total	28/56
Threads per socket/total (SMT on)	56/112
Base/max turbo frequency	2.6/3.5 GHz
L1d/L1i cache (per core)	48 KiB/32 KiB
L2 cache (per core)	1.25 MiB
L3 cache (per socket, shared)	42 MiB
NUMA nodes	2 (one per socket)
Total memory	1 TiB

For full specifications, see the manufacturer’s product page: https://www.intel.com/content/www/us/en/products/sku/212456/intel-xeon-gold-6348-processor-42m-cache-2-60-ghz/specifications.html, accessed 2 August 2026.

## Appendix G. Choosing npmax Without Prior Knowledge

The grid search yields one (n,AR) pair per combination, with *n* being the number of fitted signals. Those pairs are not monotone in *n*, so the criterion uses the cumulative minimum

(A17) $$F\left(n\right)=\min\left\{{\mathrm{AR}}_{c}\;:\;{\mathrm{np}}_{c}\leq n\right\},$$

the achievable minimum AR within a budget of *n* peaks. The elbow of that curve is taken as npmax, located by the Kneedle heuristic [28]: both axes are scaled to [0,1],

(A18) $${\tilde{n}}_{i}=\frac{n_{i}-n_{1}}{n_{m}-n_{1}},\;{\tilde{F}}_{i}=\frac{F_{i}-F_{m}}{F_{1}-F_{m}},$$

and the selected budget is the point of greatest distance from the diagonal,

(A19) $$i^{★}=\underset{i}{\arg\max}\left[\left(1-{\tilde{n}}_{i}\right)-{\tilde{F}}_{i}\right],\;\mathrm{npmax}=n_{i^{★}}.$$

AR is computed over the fitted region. Setting npmax=−1 uses the median of the per-spectrum elbows across the whole dataset, or across the training fold when used during cross-validation.

The median elbow is 1117 for the 106 AKI spectra and 988 for the 16 Blood spectra; the 2 Urine spectra give 927 and 1668.

Between 500 and 2000 peaks, the feature matrix grows from 950 to 1880 columns, while accuracy stays between 72.4 and 75.6% and AUC between 0.793 and 0.832. No setting differs from the others once the 22 comparisons are accounted for (smallest Holm-adjusted p=1.00 and 0.61; Figure A7 and Table A5).

**Table A5:** The AKI benchmark repeated at each npmax. Listed are the evenly spaced budgets from 500 to 2000 and npmax=−1, placed at the budget it resolved to on the whole cohort; across the 30 folds, it resolved to 1105–1130, the shaded band of Figure A7. Accuracy and AUC are means over those folds with one standard error. The 23 settings tested include further elbow-derived budgets, which are not listed here.

npmax	Features	Accuracy (%)	AUC
500	950	73.9 ± 1.9	0.793 ± 0.024
600	950	73.6 ± 1.8	0.806 ± 0.025
700	950	75.3 ± 2.0	0.815 ± 0.027
800	950	74.9 ± 1.8	0.814 ± 0.022
900	950	73.7 ± 1.9	0.822 ± 0.023
1000	956	74.6 ± 2.0	0.817 ± 0.026
1100	1071	74.2 ± 1.6	0.832 ± 0.021
−1	1115	73.7 ± 2.2	0.827 ± 0.025
1200	1196	73.3 ± 1.9	0.815 ± 0.029
1300	1270	72.4 ± 1.9	0.810 ± 0.027
1400	1367	74.2 ± 2.1	0.815 ± 0.028
1500	1467	74.4 ± 2.1	0.820 ± 0.029
1600	1595	75.0 ± 1.8	0.821 ± 0.027
1700	1666	74.6 ± 1.7	0.817 ± 0.027
1800	1768	73.4 ± 2.0	0.813 ± 0.026
1900	1880	72.7 ± 1.8	0.798 ± 0.027
2000	1880	73.2 ± 1.7	0.802 ± 0.027
